# Association of healthy plant-based diet-lifestyle score with obesity-related cancer incidence and mortality: a prospective cohort study in the UK Biobank

**DOI:** 10.3389/fnut.2026.1922232

**Published:** 2026-09-08

**Authors:** Jinzhou Yu, Qiang Zhang, Qianyu Zhou, Jifeng Sun, Buwei He, Zhaohan Wang, Ziqiu Guo, Mengting Liu, Mingyang Zhao, Tong Wanyan, Yulong Wan, Hua Ye, Changqing Sun

**Affiliations:** 1 School of Nursing and Health, Zhengzhou University, Zhengzhou, China; 2 College of Public Health, Zhengzhou University, Zhengzhou, China; 3 Department of Biological Sciences, De Anza College, Cupertino, CA, United States; 4 Department of Epidemiology and Biostatistics, Arnold School of Public Health, University of South Carolina, Columbia, SC, United States; 5 South Carolina SmartState Centre for Healthcare Quality, Arnold School of Public Health, University of South Carolina, Columbia, SC, United States; 6 Corona del Mar Middle and High School, Newport Beach, CA, United States; 7 School of Nursing, The University of Hong Kong, Hong Kong, Hong Kong SAR, China; 8 State Key Laboratory of Metabolic Dysregulation & Prevention and Treatment of Esophageal Cancer, Zhengzhou University, Zhengzhou, China; 9 Henan Key Laboratory of Tumor Epidemiology and Henan International Joint Laboratory of Tumor Biomarkers and Molecular Imaging, Zhengzhou, China

**Keywords:** cancer mortality, healthy plant-based diet, lifestyle score, obesity-related cancer, UK Biobank

## Abstract

**Background:**

Evidence regarding the combined influence of healthy plant-based dietary patterns and lifestyle behaviours on obesity-related cancer outcomes remains limited. We prospectively investigated the associations of the healthy plant-based diet-lifestyle (hPDI-Lifestyle) score with obesity-related cancer incidence and mortality in the UK Biobank.

**Methods:**

Among 104,713 eligible participants, 93,763 were included in the primary complete-case analyses. The hPDI-Lifestyle score integrated healthy plant-based dietary quality, physical activity, smoking status, and sleep duration. Restricted cubic spline analyses were used to examine dose–response relationships, and Cox proportional hazards models estimated hazard ratios (HRs) and 95% confidence intervals (CIs) for the three outcomes, including sex-stratified analyses. Several sensitivity analyses were conducted to assess the robustness of the findings.

**Results:**

During median follow-up periods of 16.3, 15.0, and 15.1 years, respectively, 2,258 incident obesity-related cancers, 1,095 obesity-related cancer deaths, and 6,215 all-cause deaths were documented. Higher hPDI-Lifestyle scores were associated with lower risks of obesity-related cancer mortality and all-cause mortality. For obesity-related cancer incidence, an inverse association was observed overall, whereas sex-stratified analyses showed no statistically significant association among women. Compared with participants in the lowest tertile, those in the highest tertile had lower risks of obesity-related cancer incidence (HR: 0.82, 95% CI: 0.74–0.92), obesity-related cancer-specific mortality (HR: 0.66, 95% CI: 0.56–0.77), and all-cause mortality (HR: 0.64, 95% CI: 0.60–0.68). Each 1-SD increment in the hPDI-Lifestyle score was associated with 8, 18, and 22% lower risks of obesity-related cancer incidence, obesity-related cancer-specific mortality, and all-cause mortality, respectively.

**Conclusion:**

Higher hPDI-Lifestyle scores were associated with lower risks of obesity-related cancer incidence, cancer-specific mortality, and all-cause mortality. These findings require confirmation in more representative populations and intervention studies before preventive implications can be established.

## 1 Introduction

Obesity-related cancers have become a major global public health challenge and account for a growing proportion of cancer incidence and mortality worldwide (1, 2). According to the International Agency for Research on Cancer, excess adiposity is convincingly associated with the development of several malignancies, including colorectal, liver, pancreatic, kidney, endometrial, and oesophageal cancers (3). With the continuing rise in obesity prevalence, the burden of obesity-related cancers is expected to increase substantially in the coming decades (4, 5). Therefore, identifying modifiable lifestyle factors associated with the prevention and prognosis of obesity-related cancers is of considerable clinical and public health importance.

Unhealthy lifestyle behaviours play a central role in obesity-related carcinogenesis (6). Diet quality, physical inactivity, smoking, and inadequate sleep have each been independently associated with obesity, chronic inflammation, insulin resistance, metabolic dysfunction, and impaired immune regulation, all of which contribute to cancer initiation and progression (6–9). Among dietary patterns, healthy plant-based diets characterized by higher consumption of fruits, vegetables, whole grains, legumes, and nuts have attracted increasing attention because of their beneficial effects on metabolic health and chronic disease prevention (10, 11). However, lifestyle behaviours tend to cluster together, and evaluating single behaviours in isolation may not adequately capture the combined effects of overall lifestyle patterns on cancer outcomes.

Composite lifestyle scores integrating multiple health behaviours have therefore emerged as useful tools for assessing the joint influence of modifiable factors on chronic disease risk (12). The healthy plant-based diet-lifestyle (hPDI-Lifestyle) score combines healthy plant-based dietary quality with physical activity, smoking status, and sleep duration to reflect overall adherence to a healthy lifestyle pattern according to Life’s Essential 8 metrics (13, 14). Although this score was initially developed in the context of cardiovascular disease prevention, the behavioural and metabolic pathways represented by the score are also closely linked to obesity-related cancer development and prognosis (13, 15, 16). However, the hPDI-Lifestyle score has not been validated specifically for cancer outcomes, and evidence regarding its associations with obesity-related cancer outcomes remains limited.

Therefore, we prospectively investigated the associations of the hPDI-Lifestyle score with obesity-related cancer incidence, cancer-specific mortality, and all-cause mortality using data from the UK Biobank.

## 2 Materials and methods

### 2.1 Study design and population

This prospective cohort study was based on data from the UK Biobank, a large population-based cohort that recruited 501,956 participants aged 37–73 years from 22 assessment centres across the United Kingdom between 2006 and 2010 (17). At baseline, participants provided electronic signed consent, completed touchscreen questionnaires, underwent physical examinations, and provided biological samples using standardized protocols at different centres (17). The mean intake across repeated Oxford WebQ assessments was used to calculate the healthy plant-based diet index (hPDI), which served as the dietary component of the hPDI-Lifestyle score, and the analytical baseline was defined as the date of the last valid Oxford WebQ assessment used to calculate the hPDI. For the present analysis, participants were included if they had at least two valid 24-h dietary assessments using the Oxford WebQ, had complete data required for calculation of the hPDI-Lifestyle score, and were free of obesity-related cancer at the analytical baseline. Participants who withdrew informed consent or reported implausible total energy intake (<500 or >3,500 kcal/day for women and <800 or >4,000 kcal/day for men) were also excluded. Detailed information on the UK Biobank field IDs corresponding to variables used in the present study is provided in Supplementary Table S1.

### 2.2 Healthy plant-based diet index

Dietary intake was assessed using the Oxford WebQ, a validated web-based 24-h dietary assessment questionnaire administered repeatedly between 2009 and 2012 (18). The questionnaire collected detailed information on the consumption of more than 200 food items and beverages during the previous 24 h. Food intakes were converted into gram-per-day values using standard portion sizes. To reduce within-person variation and better reflect habitual dietary intake, only participants with at least two valid dietary assessments were included, and the mean intake across repeated Oxford WebQ assessments was used for dietary analyses. Based on predefined food groups, the original healthy plant-based diet index (hPDI) was calculated using sex-specific quintiles of food-group intake (19, 20). Healthy plant food groups were positively scored from 1 to 5, whereas less healthy plant food groups and animal food groups were reverse scored from 5 to 1. For most food groups, participants were classified into sex-specific quintiles. Miscellaneous animal foods were scored separately according to consumption level, as detailed in Supplementary Table S2. The food-group scores were summed to obtain the total hPDI, with an observed range of 18 to 88.

### 2.3 Physical activity, smoking status, and sleep duration

Physical activity was assessed using self-reported moderate and vigorous physical activity collected through touchscreen questionnaires in the UK Biobank. Total physical activity was quantified using the derived variables “Metabolic Equivalent Task (MET) minutes per week for moderate activity” and “Metabolic Equivalent Task (MET) minutes per week for vigorous activity” (21, 22). Smoking status was self-reported and classified as never, former, or current smoking. Participants who never smoked received the highest score, whereas current smokers received the lowest score. Intermediate scores were assigned according to smoking cessation duration among former smokers. Sleep duration was assessed using self-reported average sleep hours per night. Optimal sleep duration (7 to <9 h/night) received the highest score, while shorter or longer sleep durations were assigned progressively lower scores.

### 2.4 Assessment of the hPDI-lifestyle score

The hPDI-Lifestyle score integrated healthy plant-based diet quality, physical activity, smoking status, and sleep duration. Following the previously published hPDI-Lifestyle scoring method by Wang et al., the total hPDI was converted into a dietary component score ranging from 0 to 100 (13). Participants with total hPDI values in the <25th, 25th–49th, 50th–74th, 75th–94th, and ≥95th percentiles were assigned 0, 25, 50, 80, and 100 points, respectively. The four component scores were equally weighted and averaged to derive the overall score, ranging from 0 to 100, with higher scores indicating greater adherence. In the primary analyses, the score was examined as tertiles and per SD increment. Detailed component scoring procedures are presented in Supplementary Table S3.

### 2.5 Ascertainment of obesity-related cancer outcomes

The primary outcome was incident obesity-related cancer. Cancer cases were identified through linkage to national cancer registries, hospital admission records, and death registries. Obesity-related cancers were defined according to established International Classification of Diseases, Tenth Revision (ICD-10) codes and included cancers of the esophagus (C15), gastric cardia (C16.0), colorectum (C18, C19.9, and C20.9), liver (C22), gallbladder (C23), pancreas (C25), kidney (C64), corpus uteri (C54 and C55), ovary (C56), thyroid (C73), meninges (C70), and multiple myeloma (C90.0) (3). Secondary outcomes included all-cause mortality and obesity-related cancer mortality. Mortality information was obtained through linkage to national death registries. Follow-up time was calculated from the date of the last valid Oxford WebQ assessment to the first occurrence of the outcome event, death, loss to follow-up, or the end of follow-up, whichever occurred first.

### 2.6 Covariates

Covariates were selected *a priori* based on previous literature and the causal framework shown in Supplementary Figure S1 (7, 15). Sex, ethnicity, education, household income, family history of cancer, total energy intake, and alcohol consumption status were considered potential confounders, whereas body mass index (BMI) and diabetes were treated as additional covariates with potentially complex causal roles. Ethnicity was categorized as White, Mixed, Asian British, Black British, and other ethnic groups. BMI was categorized as underweight, normal weight, overweight, or obesity according to standard criteria.

### 2.7 Statistical analysis

Baseline characteristics were summarized according to tertiles of the hPDI-Lifestyle score. Continuous variables were presented as means and standard deviations (SDs) or medians and interquartile ranges (IQRs), as appropriate, whereas categorical variables were described as counts and percentages. Baseline characteristics were also compared between participants in the eligible cohort and those excluded from it. Median follow-up was estimated using the reverse Kaplan–Meier method, and person-years and outcome rates per 1,000 person-years were calculated overall and across hPDI-Lifestyle score tertiles.

Cox proportional hazards regression models with attained age as the underlying time scale were used to estimate hazard ratios (HRs) and 95% confidence intervals (CIs) for the associations of the hPDI-Lifestyle score with obesity-related cancer incidence, obesity-related cancer mortality, and all-cause mortality. The lowest tertile of the hPDI-Lifestyle score was used as the reference group. Associations were additionally evaluated per SD increment in the continuous score. Three sequential models were fitted. Model 1 used attained age as the time scale and adjusted for sex. Model 2 additionally adjusted for ethnicity, education, household income, family history of cancer, total energy intake, and alcohol consumption status. Model 3 further adjusted for BMI category and diabetes and was used as the primary model. Primary analyses used complete cases, excluding participants with missing model covariates. Restricted cubic splines with four knots at the commonly recommended 5th, 35th, 65th, and 95th percentiles, corresponding to scores of 46.25, 68.75, 81.25, and 95, respectively, were used to assess nonlinearity, with the median score of 75 as the reference. Curves were restricted to the 1st–99th percentiles, and nonlinearity was evaluated using likelihood ratio tests. Sex-stratified analyses were conducted to examine potential effect modification by sex, and interaction terms between the hPDI-Lifestyle score and sex were evaluated using likelihood ratio tests. Similar approaches have been used to assess nonlinearity and subgroup heterogeneity in large population-based studies (23).

Several sensitivity analyses were performed to assess the robustness of the findings. First, participants who developed obesity-related cancer within the first 2 years of follow-up were excluded to minimize potential reverse causation. Second, the hPDI-Lifestyle score was alternatively categorized into poor, intermediate, and ideal lifestyle groups according to predefined cut-off values. Third, Fine-Gray subdistribution hazard models were conducted as competing-risk analyses for obesity-related cancer incidence and obesity-related cancer mortality, with deaths from other causes treated as competing events. Fourth, missing covariates were multiply imputed using multivariate imputation by chained equations to generate 10 imputed datasets, and Cox regression estimates were pooled using Rubin’s rules. Fifth, sex-stratified analyses were repeated after restricting the composite outcome to cancer sites occurring in both sexes. Sixth, the robustness of the findings to the score construction method was assessed using a simple count of ideal health behaviours. One point was assigned to each component meeting its ideal criterion, yielding a total score ranging from 0 to 4. Additionally, component-specific models, a mutually adjusted model including all four components, and a model using the composite score excluding smoking, with the smoking component included as a covariate, were fitted to assess whether the associations were predominantly driven by a single component. Finally, site-specific analyses were performed for cancers with at least 50 events, with female-specific cancers analysed among women only. Leave-one-cancer-site-out analyses were conducted to assess whether the composite association was driven by any single cancer site. All statistical analyses were conducted using R (version 4.4.2). A two-sided *p* value <0.05 was considered statistically significant.

## 3 Results

### 3.1 Study population

**Figure 1:**
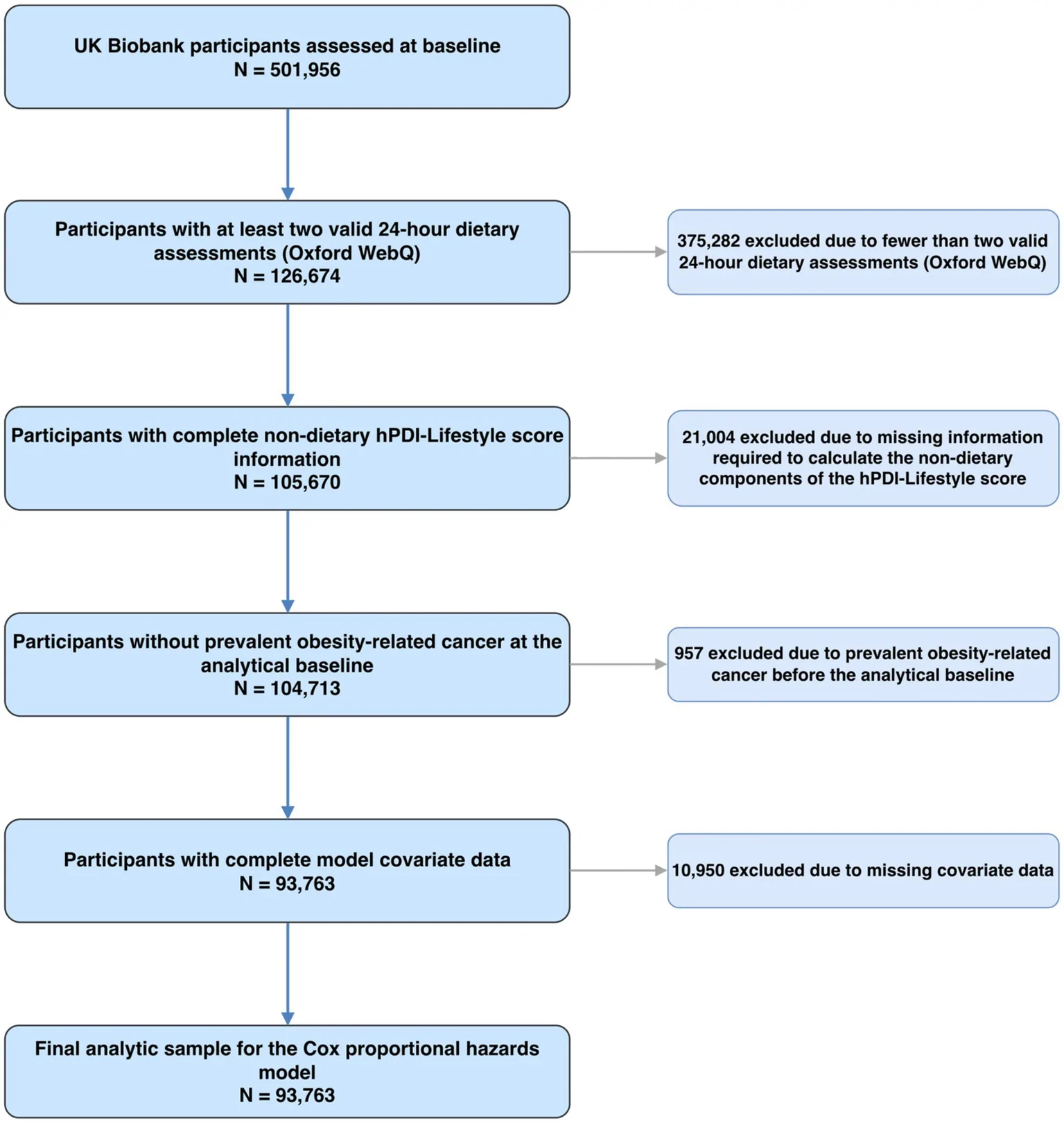
Flowchart showing selection of participants from the UK Biobank included in the main analysis.

Figure 1 shows the flowchart of participant selection for the present study. Among 501,956 UK Biobank participants, those with fewer than two valid Oxford WebQ assessments, insufficient information to calculate the non-dietary components of the hPDI-Lifestyle score, or prevalent obesity-related cancer before the analytical baseline were excluded, resulting in 104,713 participants in the eligible cohort. Of these, 10,950 participants with incomplete model covariate data were excluded from the primary complete-case analyses, leaving 93,763 participants in the primary analytical sample.

Table 1 presents the baseline characteristics of the 104,713 eligible participants stratified by hPDI-Lifestyle score tertiles. The hPDI-Lifestyle score ranged from <70 in T1 to 70–<82 in T2 and ≥82 in T3. Overall, participants were predominantly women (53.8%) and White (96.9%), with a mean age of 55.9 years. The distributions of education level, household income, lifestyle factors, and metabolic characteristics across hPDI-Lifestyle score tertiles are shown in Table 1.

**Table 1:** Baseline characteristics of participants stratified by healthy plant-based diet index-lifestyle score tertiles in the UK Biobank cohort.

Characteristics	Overall *N* = 104,713	T1 (hPDI-Lifestyle score < 70) *N* = 37,303	T2 (hPDI-Lifestyle score, 70- < 82) *N* = 38,281	T3 (hPDI-Lifestyle score, ≥82) *N* = 29,129
Sex, *N* (%)				
Women	56,300 (53.8)	19,164 (51.4)	20,828 (54.4)	16,308 (56.0)
Men	48,413 (46.2)	18,139 (48.6)	17,453 (45.6)	12,821 (44.0)
Age, years, mean (SD)	55.9 (7.9)	55.5 (7.9)	55.9 (7.9)	56.4 (7.7)
Education level, *N* (%)				
College/University degree	51,401 (49.2)	16,332 (43.9)	18,903 (49.5)	16,166 (55.6)
Other qualifications	47,360 (45.3)	18,345 (49.3)	17,295 (45.3)	11,720 (40.3)
None of the above	5,749 (5.5)	2,544 (6.8)	2,007 (5.3)	1,198 (4.1)
Missing	203 (0.2)	82 (0.2)	76 (0.2)	45 (0.2)
Ethnicity, *N* (%)				
White	101,199 (96.9)	35,918 (96.6)	37,109 (97.2)	28,172 (96.9)
Mixed	567 (0.5)	247 (0.7)	176 (0.5)	144 (0.5)
Asian British	1,270 (1.2)	422 (1.1)	434 (1.1)	414 (1.4)
Black British	811 (0.8)	360 (1.0)	276 (0.7)	175 (0.6)
Other	592 (0.6)	231 (0.6)	202 (0.5)	159 (0.5)
Missing	274 (0.3)	125 (0.3)	84 (0.2)	65 (0.2)
Average total household income before tax, *N* (%)				
<£18,000	12,510 (12.9)	5,078 (14.7)	4,315 (12.2)	3,117 (11.6)
£18,000–30,999	22,322 (23.0)	7,987 (23.1)	8,176 (23.1)	6,159 (22.9)
£31,000–51,999	28,150 (29.0)	9,989 (28.9)	10,314 (29.1)	7,847 (29.2)
£52,000–100,000	25,716 (26.5)	8,869 (25.7)	9,537 (26.9)	7,310 (27.2)
>£100,000	8,209 (8.5)	2,651 (7.7)	3,106 (8.8)	2,452 (9.1)
Missing	7,806 (7.5)	2,729 (7.3)	2,833 (7.4)	2,244 (7.7)
Alcohol consumption status, *N* (%)				
Never	2,800 (2.7)	915 (2.5)	1,048 (2.7)	837 (2.9)
Previous	2,939 (2.8)	1,226 (3.3)	932 (2.4)	781 (2.7)
Current	98,942 (94.5)	35,145 (94.3)	36,291 (94.8)	27,506 (94.4)
Missing	32 (<0.1)	17 (<0.1)	10 (<0.1)	5 (<0.1)
BMI-defined obesity category, *N* (%)				
Underweight	600 (0.6)	178 (0.5)	191 (0.5)	231 (0.8)
Normal	41,286 (39.5)	11,905 (32.0)	15,336 (40.1)	14,045 (48.3)
Overweight	42,950 (41.1)	15,669 (42.1)	15,949 (41.7)	11,332 (39.0)
Obesity	19,656 (18.8)	9,440 (25.4)	6,745 (17.6)	3,471 (11.9)
Missing	221 (0.2)	111 (0.3)	60 (0.2)	50 (0.2)
Central obesity, *N* (%)				
No	78,477 (75.0)	25,054 (67.3)	29,164 (76.2)	24,259 (83.4)
Yes	26,111 (25.0)	12,191 (32.7)	9,084 (23.8)	4,836 (16.6)
Missing	125 (0.1)	58 (0.2)	33 (<0.1)	34 (0.1)
Diabetes, *N* (%)				
No	101,041 (96.5)	35,601 (95.5)	37,148 (97.1)	28,292 (97.1)
Yes	3,637 (3.5)	1,682 (4.5)	1,123 (2.9)	832 (2.9)
Missing	35 (<0.1)	20 (<0.1)	10 (<0.1)	5 (<0.1)
Family history of cancer, *N* (%)				
No	77,826 (76.5)	27,709 (76.8)	28,429 (76.3)	21,688 (76.3)
Yes	23,959 (23.5)	8,366 (23.2)	8,851 (23.7)	6,742 (23.7)
Missing	2,928 (2.8)	1,228 (3.3)	1,001 (2.6)	699 (2.4)
Smoking status, *N* (%)				
Never	59,876 (57.2)	12,578 (33.7)	25,138 (65.7)	22,160 (76.1)
Previous	37,567 (35.9)	18,282 (49.0)	12,316 (32.2)	6,969 (23.9)
Current	7,270 (6.9)	6,443 (17.3)	827 (2.2)	0 (0.0)
Physical activity, MET min/week, median (IQR)	240.0 (0.0–960.0)	0.0 (0.0–320.0)	384.0 (0.0–960.0)	480.0 (160.0–1,200.0)
Sleep duration, hours/night, median (IQR)	7.0 (7.0–8.0)	7.0 (6.0–8.0)	7.0 (7.0–8.0)	7.0 (7.0–8.0)
Energy intake, kcal/day, median (IQR)	2,022.9 (1,724.2–2,362.2)	2,066.5 (1,760.6–2,425.4)	2,047.2 (1,749.6–2,380.0)	1,941.7 (1,657.6–2,255.2)

Missing values are reported descriptively and were not treated as separate categories in the Cox models. Sex, age, smoking status, physical activity, sleep duration, and total energy intake had no missing values.

BMI, body mass index; hPDI-Lifestyle score, healthy plant-based diet index-lifestyle score; IQR, interquartile range; MET, metabolic equivalent task; SD, standard deviation.

Compared with excluded participants (*n* = 397,243), participants in the eligible cohort (*n* = 104,713) were slightly younger (mean age, 55.9 vs. 56.7 years), more likely to have a college or university degree (49.2% vs. 28.3%), and more likely to report a higher household income. Eligible participants were also less likely to be current smokers (6.9% vs. 11.6%), have obesity (18.8% vs. 26.0%), central obesity (25.0% vs. 32.6%), or diabetes (3.5% vs. 5.3%) (Supplementary Table S11). During median follow-up periods of 16.3, 15.0, and 15.1 years, 2,258 incident obesity-related cancers, 1,095 obesity-related cancer deaths, and 6,215 all-cause deaths were documented over 1,489,604, 1,392,855, and 1,392,855 person-years, respectively. Events, person-years, and rates per 1,000 person-years across hPDI-Lifestyle score tertiles are presented in Supplementary Table S10.

### 3.2 Dose–response associations between the hPDI-lifestyle score and study outcomes

**Figure 2:**
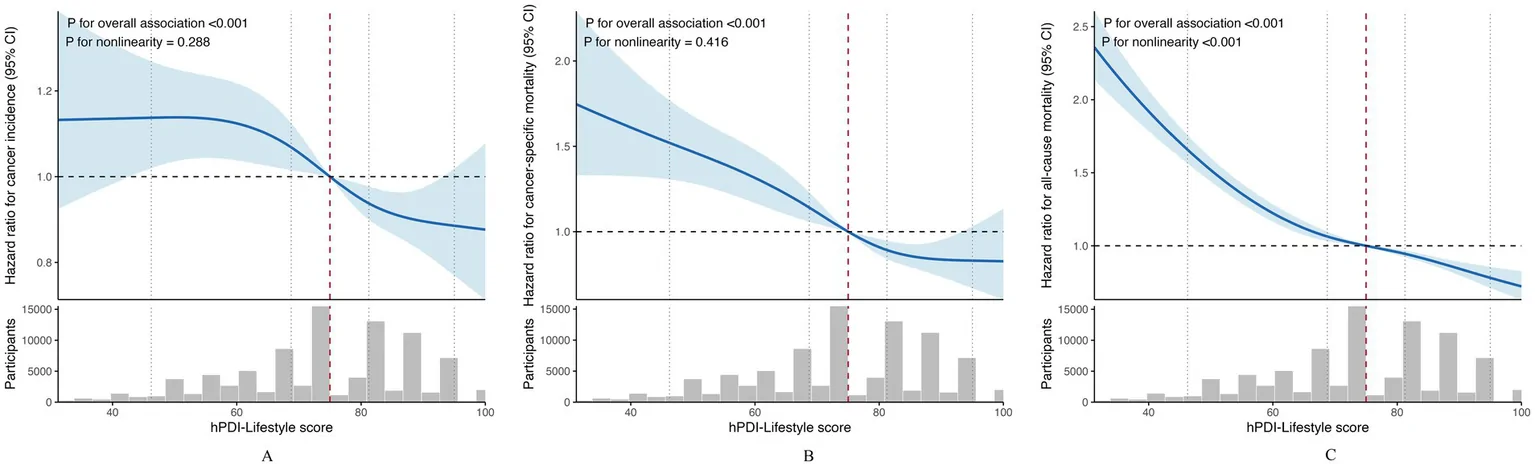
Dose–response associations between the continuous hPDI-Lifestyle score and study outcomes (based on restricted cubic spline analyses). **(A)** Incident obesity-related cancer. **(B)** Obesity-related cancer mortality. **(C)** All-cause mortality. hPDI-Lifestyle score, healthy plant-based diet index-lifestyle score. Grey dotted lines indicate knots at the 5th, 35th, 65th, and 95th percentiles, and the red dashed line indicates the median reference value of 75. Curves are displayed from the 1st to the 99th percentile; estimates at the distributional extremes should be interpreted cautiously because of sparse data. Models were adjusted for BMI category, diabetes, total energy intake, alcohol consumption status, sex, ethnicity, education level, family history of cancer, and household income.

Figure 2 presents the restricted cubic spline associations between the continuous hPDI-Lifestyle score and the study outcomes. Significant overall associations were observed for all three outcomes (all P for overall association < 0.001). No statistical evidence of nonlinearity was detected for obesity-related cancer incidence (*p* = 0.288) or cancer-specific mortality (*p* = 0.416), whereas evidence of nonlinearity was detected for all-cause mortality (*p* < 0.001), with a steeper decline in the hazard ratio at lower score ranges followed by a more gradual decline at higher scores.

### 3.3 Associations of the hPDI-lifestyle score with study outcomes

**Figure 3:**
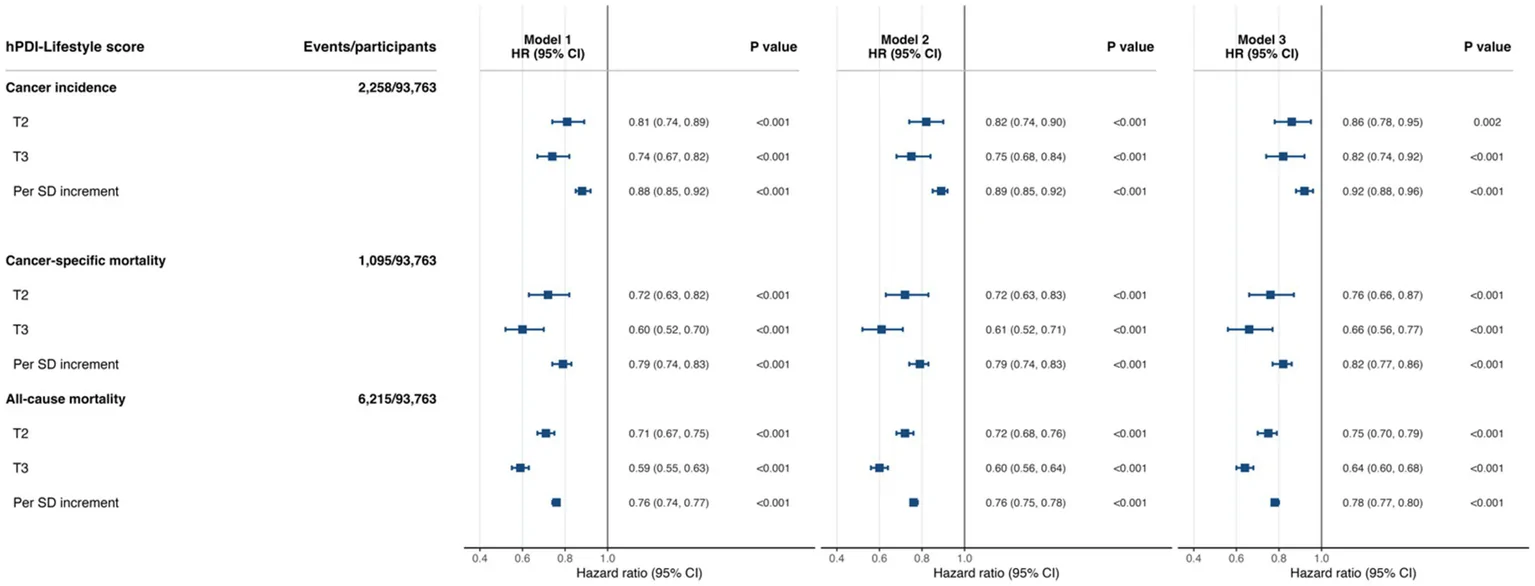
Associations of the healthy plant-based diet index-lifestyle score with obesity-related cancer incidence, obesity-related cancer mortality, and all-cause mortality. Model 1 used attained age as the underlying time scale and adjusted for sex; Model 2 additionally adjusted for ethnicity, education, household income, family history of cancer, total energy intake, and alcohol consumption status; Model 3 further adjusted for BMI category and diabetes. hPDI-lifestyle score, healthy plant-based diet index-lifestyle score; SD, standard deviation; CI, confidence interval.

Figure 3 shows the associations of the hPDI-Lifestyle score with obesity-related cancer incidence, obesity-related cancer mortality, and all-cause mortality across three sequential models. Inverse associations were observed in all models, although the estimates were modestly attenuated after further adjustment for BMI and diabetes. In the fully adjusted model, the HRs per SD increment in the hPDI-Lifestyle score were 0.92 (95% CI: 0.88–0.96) for obesity-related cancer incidence, 0.82 (95% CI: 0.77–0.86) for obesity-related cancer-specific mortality, and 0.78 (95% CI: 0.77–0.80) for all-cause mortality. No substantial violations of the proportional hazards assumption were identified for the primary exposure variables based on Schoenfeld residual tests.

### 3.4 Sex-stratified analyses

**Figure 4:**
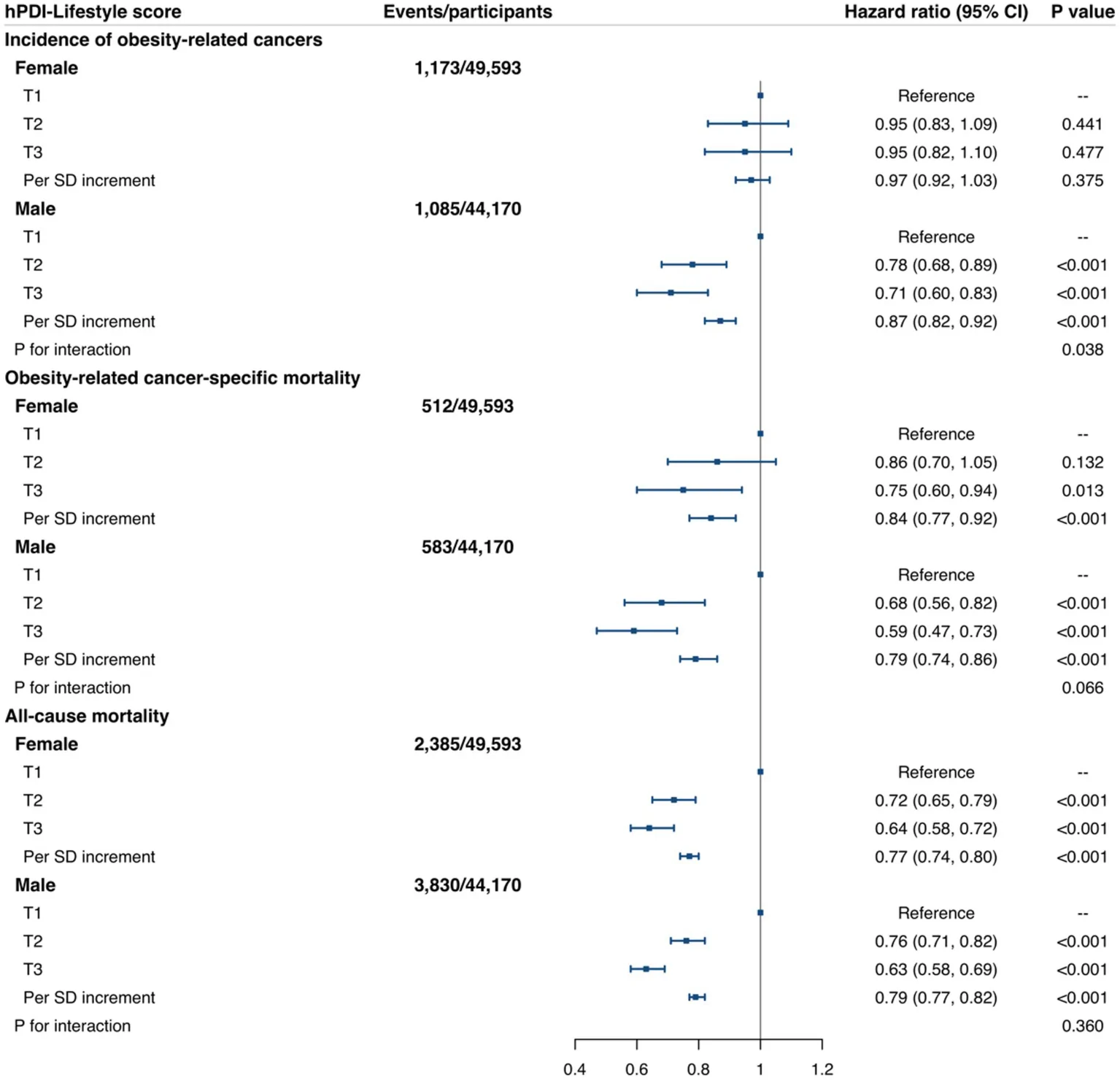
Sex-stratified associations of the healthy plant-based diet index-lifestyle score with obesity-related cancer incidence, obesity-related cancer mortality, and all-cause mortality. Models were adjusted for BMI category, diabetes, total energy intake, alcohol consumption status, ethnicity, education, family history of cancer, and household income. hPDI-Lifestyle score, Healthy plant-based diet index-lifestyle score; SD, Standard deviation; CI, Confidence interval.

Figure 4 shows the sex-stratified analyses. For obesity-related cancer incidence, the inverse association with the hPDI-Lifestyle score appeared stronger among men than women. Among men, participants in the highest tertile had a 29% lower risk of obesity-related cancer incidence (HR: 0.71, 95% CI: 0.60–0.83), whereas no statistically significant association was observed among women (HR: 0.95, 95% CI: 0.82–1.10; P for interaction = 0.038). In contrast, the associations of the hPDI-Lifestyle score with obesity-related cancer mortality and all-cause mortality were generally similar across sexes, with no significant interaction observed. In sensitivity analyses, the sex interaction for obesity-related cancer incidence was no longer statistically significant after excluding early cases or restricting the composite outcome to cancer sites occurring in both sexes.

### 3.5 Sensitivity and additional analyses

Overall, the main associations remained similar across sensitivity analyses. After excluding obesity-related cancer cases occurring within the first 2 years of follow-up, higher hPDI-Lifestyle scores remained significantly associated with lower risks of obesity-related cancer incidence, obesity-related cancer mortality, and all-cause mortality (Supplementary Table S4). However, the previously observed sex interaction for obesity-related cancer incidence was no longer statistically significant (Supplementary Table S8). Similar inverse associations were also observed using an alternative categorization of the hPDI-Lifestyle score (Supplementary Tables S5, S7). Fine-Gray competing-risk analyses yielded similar results for obesity-related cancer incidence (HR per SD increment: 0.94, 95% CI: 0.90–0.98) and obesity-related cancer mortality (HR per SD increment: 0.88, 95% CI: 0.83–0.92) (Supplementary Table S6). Multiple-imputation analyses were also consistent with the complete-case analyses. The HRs per SD increment were 0.91 (95% CI: 0.88–0.95) for obesity-related cancer incidence, 0.81 (95% CI: 0.76–0.85) for obesity-related cancer mortality, and 0.78 (95% CI: 0.76–0.80) for all-cause mortality (Supplementary Table S9).

The distributions of the four component scores are presented in Supplementary Table S12. The component scores were weakly correlated with each other, with pairwise Spearman correlation coefficients ranging from −0.007 to 0.079 (Supplementary Table S13). In the simple-count analysis, each additional ideal health behaviour was associated with a lower risk of obesity-related cancer incidence (HR: 0.89, 95% CI: 0.85–0.93). Compared with participants with no ideal health behaviours, those with three or four ideal behaviours had lower risks of obesity-related cancer incidence, whereas the associations for one or two ideal behaviours were not statistically significant (Supplementary Table S14).

Component-specific and mutually adjusted associations for hPDI, physical activity, smoking, and sleep are presented in Supplementary Table S15. After mutual adjustment, physical activity and smoking were inversely associated with all three outcomes. The hPDI component was inversely associated with obesity-related cancer-specific mortality and all-cause mortality but not with obesity-related cancer incidence, whereas sleep was inversely associated only with all-cause mortality. The score excluding smoking remained inversely associated with obesity-related cancer incidence (HR per SD increment: 0.94, 95% CI: 0.90–0.98), obesity-related cancer-specific mortality (HR: 0.88, 95% CI: 0.83–0.94), and all-cause mortality (HR: 0.86, 95% CI: 0.84–0.88).

Site-specific estimates varied across cancer sites. Inverse associations were observed for oesophageal and pancreatic cancers, while the continuous per-SD estimates were inverse for colorectal and liver cancers. No clear inverse associations were observed for kidney, corpus uteri, ovarian, thyroid, or multiple myeloma (Supplementary Table S16). Nevertheless, leave-one-cancer-site-out analyses yielded similar estimates after excluding each site in turn, with HRs per SD increment ranging from 0.89 to 0.93 (Supplementary Table S17).

## 4 Discussion

In this large prospective UK Biobank cohort, higher hPDI-Lifestyle scores were associated with lower risks of obesity-related cancer incidence, obesity-related cancer mortality, and all-cause mortality in both tertile-based and continuous analyses. These findings extend previous evidence by evaluating a multidimensional score that integrates healthy plant-based dietary patterns with physical activity, smoking, and sleep behaviours in relation to obesity-related cancer outcomes (13, 15, 16). The inverse association with cancer incidence observed using a simple count of ideal health behaviours was broadly consistent with the main analysis, supporting the robustness of the findings to an alternative score construction method. Component-specific analyses indicated that individual behaviours contributed differently across outcomes and that the observed associations were not solely driven by a single component.

Restricted cubic spline analyses detected no statistical evidence of nonlinearity for obesity-related cancer incidence or cancer-specific mortality, whereas a nonlinear association was observed for all-cause mortality. For all-cause mortality, the risk declined more steeply at lower score ranges and more gradually at higher score levels. This pattern suggests that differences in all-cause mortality risk were greater across lower score ranges than across higher score ranges. A similar nonlinear association with a threshold effect has been reported between the Life’s Essential 8 score and all-cause mortality among individuals with cardiovascular disease (24).

Previous studies have reported inverse associations between healthy plant-based diets or composite lifestyle scores and risks of overall cancer incidence and mortality, although evidence specifically focused on obesity-related cancers remains limited (11, 25–28). Several prospective cohort studies have shown that healthier plant-based dietary patterns are associated with lower risks of colorectal cancer, gastrointestinal cancers, and cancer mortality (11, 26, 29). Similarly, studies evaluating combined lifestyle behaviours, including smoking, physical activity, and dietary quality, have demonstrated lower risks of cancer-related outcomes among individuals with healthier lifestyle profiles (27, 30). Compared with previous reports, the associations observed in the present study were relatively moderate for obesity-related cancer incidence but stronger for mortality outcomes (11, 30). One possible explanation is that obesity-related cancers are particularly influenced by metabolic and inflammatory dysfunction, which may be more comprehensively captured by the integrated hPDI-Lifestyle score than by dietary indices alone (31, 32). In addition, the inclusion of smoking and physical activity components may strengthen associations with mortality outcomes because these behaviours substantially influence systemic health and long-term survival (30).

Sex-stratified analyses initially suggested a stronger inverse association between the hPDI-Lifestyle score and obesity-related cancer incidence among men. However, the interaction was no longer statistically significant after excluding cases occurring within the first 2 years of follow-up or after restricting the composite outcome to cancer sites occurring in both sexes. The initial finding may therefore have been influenced by early events, baseline health differences, or differences in cancer-site composition between women and men (33). Accordingly, these results do not establish a true sex difference in the association.

Site-specific estimates varied across individual cancers, and inverse associations were not uniformly observed for all cancer sites. Previous studies of combined lifestyle scores have similarly reported site-specific heterogeneity, with healthier lifestyles associated with lower risks of colorectal, lung, endometrial, pancreatic, and kidney cancers, but not consistently with ovarian cancer (30, 34). Nevertheless, the leave-one-cancer-site-out analyses yielded similar estimates after excluding each site in turn, suggesting that the association with the composite outcome was not disproportionately driven by a single cancer site. Given the limited number of events for several cancer sites and the multiple site-specific comparisons, these findings should be interpreted cautiously.

The observed associations are biologically plausible given the established role of metabolic dysfunction in obesity-related carcinogenesis. Obesity-related cancers are strongly linked to insulin resistance, chronic low-grade inflammation, altered adipokine signalling, oxidative stress, and impaired immune regulation (31, 35). Healthy plant-based diets are rich in dietary fibre, antioxidants, and polyphenols, which may improve insulin sensitivity and reduce systemic inflammatory burden (32). Physical activity additionally improves metabolic regulation and immune function, whereas smoking and insufficient sleep are known to exacerbate oxidative stress and inflammatory pathways (36). Therefore, the hPDI-Lifestyle score may better reflect overall metabolic health status than isolated behavioural factors, potentially explaining the inverse associations across both incidence and mortality outcomes. In the broader context of cancer control, lifestyle-based risk assessment primarily supports population-level prevention and risk stratification, whereas artificial intelligence-assisted endoscopy and imaging-based approaches facilitate early cancer detection and precision diagnosis (37, 38). These approaches represent complementary components of cancer control rather than interchangeable strategies.

This study has several strengths. It was based on a large prospective cohort from the UK Biobank, which provided sufficient statistical power to examine the associations of the hPDI-Lifestyle score with obesity-related cancer incidence, obesity-related cancer mortality, and all-cause mortality. In addition, the use of repeated Oxford WebQ assessments helped better capture habitual dietary intake, and the findings were further supported by sex-stratified, spline, and sensitivity analyses. Nevertheless, we also acknowledge several limitations. First, the observational design precludes causal inference, and residual and unmeasured confounding cannot be completely excluded despite multivariable adjustment. Moreover, adjustment for BMI and diabetes may have attenuated associations operating through metabolic pathways. Second, lifestyle behaviours were primarily assessed at baseline and may have changed during follow-up. These self-reported measures were also susceptible to measurement error. Although the equal-weighted score followed a published framework, some exposure misclassification is possible, and its validity for cancer outcomes requires further evaluation. Third, the hPDI-Lifestyle score did not include alcohol consumption as a component, although alcohol intake was adjusted for in the multivariable models to account for its potential confounding effect. Reverse causation cannot be entirely excluded despite excluding early cancer cases. Furthermore, because the definition of obesity-related cancers included female-specific malignancies such as endometrial and ovarian cancers, differences in outcome composition between men and women may also have influenced the observed sex-specific associations. The heterogeneity of the included cancer sites and uncertainty surrounding the sex interaction warrant cautious interpretation. Finally, UK Biobank participants are predominantly of European ancestry and generally healthier than the broader population, which may limit the generalizability of the findings. The substantial participant exclusions may have further contributed to selection bias. In addition, the observed relative associations do not directly represent the absolute benefits achievable through lifestyle intervention.

## 5 Conclusion

In conclusion, higher hPDI-Lifestyle scores were associated with lower risks of obesity-related cancer incidence, cancer-specific mortality, and all-cause mortality in the overall analyses, although no statistically significant association with cancer incidence was observed among women. The sex-specific difference was not robust in sensitivity analyses. Confirmation in more representative populations and intervention studies is needed before preventive implications can be established.

## Data Availability

The datasets presented in this article are not readily available because the data used in this study were obtained from the UK Biobank under approved application and are not publicly available from the corresponding author. Access to these data is subject to UK Biobank policies and can be obtained by qualified researchers through a formal application to the UK Biobank. Requests to access the datasets should be directed to http://www.ukbiobank.ac.uk.

## References

[1] Pati S Irfan W Jameel A Ahmed S Shahid RK. Obesity and cancer: a current overview of epidemiology, pathogenesis, outcomes, and management. Cancer. (2023) 15:485. doi: 10.3390/cancers15020485, 36672434 PMC9857053

[2] Islami F Goding Sauer A Miller KD Siegel RL Fedewa SA Jacobs EJ . Proportion and number of cancer cases and deaths attributable to potentially modifiable risk factors in the United States. CA Cancer J Clin. (2018) 68:31–54. doi: 10.3322/caac.21440, 29160902

[3] Lauby-Secretan B Scoccianti C Loomis D Grosse Y Bianchini F Straif K. Body fatness and cancer—viewpoint of the IARC working group. N Engl J Med. (2016) 375:794–8. doi: 10.1056/NEJMsr1606602, 27557308 PMC6754861

[4] Arnold M Pandeya N Byrnes G Renehan AG Stevens GA Ezzati M . Global burden of cancer attributable to high body-mass index in 2012: a population-based study. Lancet Oncol. (2015) 16:36–46. doi: 10.1016/S1470-2045(14)71123-4, 25467404 PMC4314462

[5] Sung H Ferlay J Siegel RL Laversanne M Soerjomataram I Jemal A . Global cancer statistics 2020: GLOBOCAN estimates of incidence and mortality worldwide for 36 cancers in 185 countries. CA Cancer J Clin. (2021) 71:209–49. doi: 10.3322/caac.21660, 33538338

[6] Kerr J Anderson C Lippman SM. Physical activity, sedentary behaviour, diet, and cancer: an update and emerging new evidence. Lancet Oncol. (2017) 18:e457–71. doi: 10.1016/S1470-2045(17)30411-4, 28759385 PMC10441558

[7] Park J Morley TS Kim M Clegg DJ Scherer PE. Obesity and cancer—mechanisms underlying tumour progression and recurrence. Nat Rev Endocrinol. (2014) 10:455–65. doi: 10.1038/nrendo.2014.94, 24935119 PMC4374431

[8] Hecht SS. Tobacco smoke carcinogens and lung cancer. J Natl Cancer Inst. (1999) 91:1194–210. doi: 10.1093/jnci/91.14.1194, 10413421

[9] Irwin MR. Why sleep is important for health: a psychoneuroimmunology perspective. Annu Rev Psychol. (2015) 66:143–72. doi: 10.1146/annurev-psych-010213-115205, 25061767 PMC4961463

[10] Córdova R Kim J Thompson AS Noh H Shah S Dahm CC . Plant-based dietary patterns and age-specific risk of multimorbidity of cancer and cardiometabolic diseases: a prospective analysis. Lancet Healthy Longev. (2025) 6:100742. doi: 10.1016/j.lanhl.2025.100742, 40845891 PMC12408430

[11] Thompson AS Tresserra-Rimbau A Karavasiloglou N Jennings A Cantwell M Hill C . Association of healthful plant-based diet adherence with risk of mortality and major chronic diseases among adults in the UK. JAMA Netw Open. (2023) 6:e234714. doi: 10.1001/jamanetworkopen.2023.4714, 36976560 PMC10051114

[12] Li Y Schoufour J Wang DD Dhana K Pan A Liu X . Healthy lifestyle and life expectancy free of cancer, cardiovascular disease, and type 2 diabetes: prospective cohort study. BMJ. (2020) 368:l6669. doi: 10.1136/bmj.l6669, 31915124 PMC7190036

[13] Wang XJ Voortman T Bos D Kavousi M Ghanbari M Conrad N . Association between healthy plant-based diet-lifestyle (hPDI-lifestyle) score and incidence of coronary heart disease, and effect modification by genetic predisposition: a prospective analysis in a population-based cohort. Lancet Reg Health Eur. (2026) 64:101619. doi: 10.1016/j.lanepe.2026.101619 PMC12936782 41767894

[14] Lloyd-Jones DM Allen NB Anderson CA Black T Brewer LC Foraker RE . Life’s essential 8: updating and enhancing the American Heart Association’s construct of cardiovascular health: a presidential advisory from the American Heart Association. Circulation. (2022) 146:e18–43. doi: 10.1161/CIR.0000000000001078, 35766027 PMC10503546

[15] Rassy N Van Straaten A Carette C Hamer M Rives-Lange C Czernichow S. Association of healthy lifestyle factors and obesity-related diseases in adults in the UK. JAMA Netw Open. (2023) 6:e2314741. doi: 10.1001/jamanetworkopen.2023.14741, 37234008 PMC10220514

[16] Vinke PC Navis G Kromhout D Corpeleijn E. Associations of diet quality and all-cause mortality across levels of cardiometabolic health and disease: a 7.6-year prospective analysis from the Dutch lifelines cohort. Diabetes Care. (2021) 44:1228–35. doi: 10.2337/dc20-2709, 33963020

[17] Sudlow C Gallacher J Allen N Beral V Burton P Danesh J . UK biobank: an open access resource for identifying the causes of a wide range of complex diseases of middle and old age. PLoS Med. (2015) 12:e1001779. doi: 10.1371/journal.pmed.1001779, 25826379 PMC4380465

[18] Liu B Young H Crowe FL Benson VS Spencer EA Key TJ . Development and evaluation of the Oxford WebQ, a low-cost, web-based method for assessment of previous 24 h dietary intakes in large-scale prospective studies. Public Health Nutr. (2011) 14:1998–2005. doi: 10.1017/S1368980011000942, 21729481

[19] Schaefer E Barbaresko J Roden M Kuss O Schlesinger S. Plant-based dietary patterns associated with reduced risk of all-cause mortality in diabetes subgroups: a prospective cohort study from the UK biobank. Diabetes Care. (2025) 48:1850–60. doi: 10.2337/dc25-0344, 40876058 PMC12451828

[20] Satija A Bhupathiraju SN Rimm EB Spiegelman D Chiuve SE Borgi L . Plant-based dietary patterns and incidence of type 2 diabetes in US men and women: results from three prospective cohort studies. PLoS Med. (2016) 13:e1002039. doi: 10.1371/journal.pmed.1002039, 27299701 PMC4907448

[21] Hildebrand M Van Hees VT Hansen BH Ekelund U. Age group comparability of raw accelerometer output from wrist-and hip-worn monitors. Med Sci Sports Exerc. (2014) 46:1816–24. doi: 10.1249/MSS.0000000000000289, 24887173

[22] Rowlands AV Plekhanova T Yates T Mirkes EM Davies M Khunti K . Providing a basis for harmonization of accelerometer-assessed physical activity outcomes across epidemiological datasets. J Meas Phys Behav. (2019) 2:131–42. doi: 10.1123/jmpb.2018-0073

[23] Ning Z Xiao J. The positive association between blood urea nitrogen and bone mineral density in US adults—the NHANES 2011-2018. Nutr Hosp. (2011) 43:402–9. doi: 10.20960/nh.05893, 41400506

[24] Yang Y Wang Y Mao Y Zhu F Zhang M Pan M . Association of life’s essential 8 with mortality among the individuals with cardiovascular disease. Sci Rep. (2024) 14:18520. doi: 10.1038/s41598-024-69603-0, 39122961 PMC11315880

[25] Wang Y Liu B Han H Hu Y Zhu L Rimm EB . Associations between plant-based dietary patterns and risks of type 2 diabetes, cardiovascular disease, cancer, and mortality–a systematic review and meta-analysis. Nutr J. (2023) 22:46. doi: 10.1186/s12937-023-00877-2, 37789346 PMC10548756

[26] Liu F Lv Y Peng Y Qiao Y Wang P Si C . Plant-based dietary patterns, genetic predisposition and risk of colorectal cancer: a prospective study from the UK biobank. J Transl Med. (2023) 21:669. doi: 10.1186/s12967-023-04522-8, 37759216 PMC10536761

[27] Wu E Ni J-T Zhu Z-H Xu H-Q Tao L Xie T. Association of a healthy lifestyle with all-cause, cause-specific mortality and incident cancer among individuals with metabolic syndrome: a prospective cohort study in UK biobank. Int J Environ Res Public Health. (2022) 19:9936. doi: 10.3390/ijerph19169936, 36011568 PMC9408492

[28] Ding J Schöttker B Brenner H Hoffmeister M. Thirteen simple lifestyle scores and risk of cancer, cardiovascular disease, diabetes, and mortality: prospective cohort study in the UK biobank. Int J Cancer. (2025) 157:2495–505. doi: 10.1002/ijc.70064, 40704764 PMC12541561

[29] Cai Y Hong C Han J Fan L Xiao X Xiao J . Healthy dietary patterns, genetic risk, and gastrointestinal cancer incident risk: a large-scale prospective cohort study. Am J Clin Nutr. (2024) 119:406–16. doi: 10.1016/j.ajcnut.2023.11.015, 38042409

[30] Zhang Y-B Pan X-F Chen J Cao A Zhang Y-G Xia L . Combined lifestyle factors, incident cancer, and cancer mortality: a systematic review and meta-analysis of prospective cohort studies. Br J Cancer. (2020) 122:1085–93. doi: 10.1038/s41416-020-0741-x, 32037402 PMC7109112

[31] Winn M Karra P Haaland B Doherty JA Summers SA Litchman ML . Metabolic dysfunction and obesity-related cancer: results from the cross-sectional National Health and nutrition examination survey. Cancer Med. (2023) 12:606–18. doi: 10.1002/cam4.4912, 35719035 PMC9844618

[32] Thomas MS Calle M Fernandez ML. Healthy plant-based diets improve dyslipidemias, insulin resistance, and inflammation in metabolic syndrome. A narrative review. Adv Nutr. (2023) 14:44–54. doi: 10.1016/j.advnut.2022.10.002, 36811593 PMC10103000

[33] Strain T Wijndaele K Sharp SJ Dempsey PC Wareham N Brage S. Impact of follow-up time and analytical approaches to account for reverse causality on the association between physical activity and health outcomes in UK biobank. Int J Epidemiol. (2020) 49:162–72. doi: 10.1093/ije/dyz212, 31651957 PMC7124507

[34] Chen SL Braaten T Borch KB Ferrari P Sandanger TM Nøst TH. Combined lifestyle behaviors and the incidence of common cancer types in the Norwegian women and Cancer study (NOWAC). Clin Epidemiol. (2021) 13:721–34. doi: 10.2147/clep.s312864, 34429658 PMC8378914

[35] da Cunha Junior AD Carrilho LAO Nunes Filho PRS Cantini L Vidal L Mendes MCS . The role of metabolic inflammation and insulin resistance in obesity-associated carcinogenesis–a narrative review. Onco. (2025) 5:47. doi: 10.3390/onco5040047

[36] Knutson KL Spiegel K Penev P Van Cauter E. The metabolic consequences of sleep deprivation. Sleep Med Rev. (2007) 11:163–78. doi: 10.1016/j.smrv.2007.01.002, 17442599 PMC1991337

[37] Ning Z-X Xiao J-J Zhou Z-X. Artificial intelligence-assisted endoscopy in the detection of early gastrointestinal cancer: progress, challenges, and future directions. World J Gastroenterol. (2026) 32:115990. doi: 10.3748/wjg.v32.i12.115990

[38] Zhou Z-X Xiao J-J Ning Z-X. Advances in artificial intelligence for imaging-based diagnosis of hepatocellular carcinoma. World J Hepatol. (2026) 18:116233. doi: 10.4254/wjh.v18.i3.116233.

