# Supplementary material for: Association of healthy plant-based diet-lifestyle score with obesity-related cancer incidence and mortality: a prospective cohort study in the UK Biobank
Source: Front Nutr. 2026 Sep 8;13:1922232. doi: 10.3389/fnut.2026.1922232 (PMC13593403; doi:10.3389/fnut.2026.1922232)
Supplement: Supplementary file 1 [file Table_1.docx]

Supplementary Material

[Supplementary Table S1. Correspondence Between Variables Used in the Present Study and UK Biobank Data Field IDs ^a^.	1](#_Toc236778769)

[Supplementary Table S2. Food group classification and calculation method for healthy Plant-Based Diet Index ^a^.	3](#_Toc236778770)

[Supplementary Table S3. Calculation method for healthy plant-based diet-lifestyle score ^a^.	4](#_Toc236778771)

[Supplementary Figure S1. Directed acyclic graph illustrating the causal framework for covariate selection in the association between the hPDI-Lifestyle score and obesity-related cancer risk ^a^.	5](#_Toc236778772)

[Supplementary Table S4. Associations of hPDI-Lifestyle score with incidence, all-cause mortality, and cause-specific mortality of obesity-related cancers after excluding cases diagnosed within the first 2 years of follow-up.	5](#_Toc236778773)

[Supplementary Table S5. Associations of the hPDI-Lifestyle score with obesity-related cancer incidence, cause-specific mortality, and all-cause mortality using an alternative categorical classification of the hPDI-Lifestyle score ^a^.	6](#_Toc236778774)

[Supplementary Table S6. Competing-risk analyses for the associations of the hPDI-Lifestyle score with incidence and cause-specific mortality of obesity-related cancers using Fine-Gray subdistribution hazard models.	6](#_Toc236778775)

[Supplementary Table S7. Associations of the hPDI-Lifestyle score with obesity-related cancer incidence, cause-specific mortality, and all-cause mortality stratified by sex using an alternative categorical classification of the hPDI-Lifestyle score.	6](#_Toc236778776)

[Supplementary Table S8. Sex-stratified associations with obesity-related cancer incidence, cancer-specific mortality, and all-cause mortality after excluding events occurring within the first two years.	7](#_Toc236778777)

[Supplementary Table S9. Associations of the hPDI-Lifestyle score with obesity-related cancer incidence, obesity-related cancer-specific mortality, and all-cause mortality after multiple imputation.	8](#_Toc236778778)

[Supplementary Table S10. Numbers of participants, events, person-years, and event rates per 1,000 person-years across hPDI-Lifestyle score tertiles for obesity-related cancer incidence, obesity-related cancer mortality, and all-cause mortality ^a^.	9](#_Toc236778779)

[Supplementary Table S11. Comparison of baseline characteristics between participants included in and excluded from the eligible cohort ^a^.	9](#_Toc236778780)

[Supplementary Table S12. Distribution of the four component scores of the hPDI-Lifestyle score ^a^.	10](#_Toc236778781)

[Supplementary Table S14. Association between the number of ideal health behaviours and obesity-related cancer incidence.	10](#_Toc236778782)

[Supplementary Table S15. Component-specific, mutually adjusted, and smoking-excluded associations of the hPDI-Lifestyle score components with obesity-related cancer incidence and mortality outcomes.	11](#_Toc236778783)

[Supplementary Table S16. Site-specific associations of the hPDI-Lifestyle score with obesity-related cancer incidence.	11](#_Toc236778784)

[Supplementary Table S17. Leave-one-cancer-site-out associations of the hPDI-Lifestyle score with obesity-related cancer incidence.	12](#_Toc236778785)

[Supplementary Table S18. Sex-stratified associations of the hPDI-Lifestyle score with incidence and cancer-specific mortality of obesity-related cancers occurring in both sexes.	13](#_Toc236778786)

Appendix content from the manuscript is provided below as supplementary tables and figure.

Supplementary Table S1. Correspondence Between Variables Used in the Present Study and UK Biobank Data Field IDs ^a^.

| Variable Used in This Study | UK Biobank Field ID | Description |
| --- | --- | --- |
| Oxford WebQ 24-hour dietary recall | 26076, 26071, 26105, 26077, 26074, 26114, 26072, 26089, 26090, 26091, 26092, 26093, 26094, 26065, 26098, 26144, 26147, 26143, 26123, 26146, 26125, 26115, 26106, 26107, 26108, 26101, 26136, 26086, 26137, 26110, 26081, 26082, 26141, 26142, 26095, 26079, 26078, 26075, 26073, 26068, 26113, 26120, 26119, 26118, 26080, 26154, 26140, 26084, 26085, 26064, 26127, 26126, 26062, 26063, 26099, 26103, 26087, 26096, 26102, 26150, 26131, 26133, 26088, 26109, 26149, 26132, 26070, 26066, 26100, 26104, 26117, 26121, 26122, 26069, 26116, 26128 | Defined-instances run from 0 to 4. |
| Total energy intake | 26002 | Estimated food nutrients yesterday, and Kj were recalculated to kcal  (KJ/4.184:KCAL). |
| Physical activity | 22038, 22039 | Metabolic Equivalent Task (MET) minutes per week for moderate/vigorous activity. |
| Smoking status | 20116 | Never, former, or current. |
| Quit years for formal smokers | 22507 | Age of stopping smoking.  Quit years were calculated by subtracting the self-reported age at smoking cessation at baseline from the participant’s age at baseline assessment among former smokers. |
| Sleep duration | 1160 | Average sleep duration per 24 hours. |
| Body mass index (BMI) | 21001 | BMI measured at baseline assessment. |
| Age at recruitment | 21022 | Age when attended assessment centre. |
| Sex | 31 | Biological sex. |
| Ethnicity | 21000 | Self-reported ethnic background. |
| Education level | 6138 | Highest educational qualification. |
| Average household income | 738 | Average total household income before tax |
| Alcohol drinking status | 20117 | Never, former, or current. |
| Diabetes | 2443 / 20002 | Doctor-diagnosed diabetes or self-reported diabetes. |
| Family history of cancer | 20110 / 20107 | Family history of cancer in first-degree relatives. |
| Incident obesity-related cancer | 40006 | ICD-10 cancer diagnosis records. ICD-10 codes C15 oesophagus, C16.0 gastric cardia, C18 colon, C19.9 and C20.9 rectum, C22 liver, C23 gallbladder, C25 pancreas, C54 and C55 corpus uteri, C56 ovary, C64 kidney, C70 malignant neoplasm of meninges, C73 thyroid, C90.0 multiple myeloma |
| Date of death | 40000 | Date of death. |
| Primary cause of death | 40001 | ICD-10 primary cause of death. |

^a^UK Biobank field IDs refer to the UK Biobank Showcase.

Abbreviations: BMI, body mass index; hPDI, healthy plant-based diet index; ICD-10, International Classification of Diseases, Tenth Revision; MET, metabolic equivalent task.

Supplementary Table S2. Food group classification and calculation method for healthy Plant-Based Diet Index ^a^.

| Category | Food group | Food groups available in the UK Biobank cohort |
| --- | --- | --- |
| Healthy foods | Whole grains | Bran cereal; Mixed bread, brown and seeded; Muesli; Oat cereal (non-sugar); Wholemeal bread; Whole-meal pasta; Brown rice and other wholegrains |
|  | Fruit | Apples and pears; Berries; Citrus; Dried fruit; Other fruit |
|  | Vegetables | Allium vegetables; Green leafy/cabbages; Vegetable dips and side dishes; Tomatoes; Raw salad; Other vegetables (mushrooms, fruiting and mixed vegetables); Root vegetables; Peas and sweetcorn |
|  | Nuts | Nut-based spreads; Unsalted nuts and seeds; Salted nuts and seeds |
|  | Legumes | Legumes and pulses; Meat substitutes-soy |
|  | Vegetable oils | Olive oil (drizzling/dunking); |
|  | Tea & Coffee | Coffee; Coffee (decaffeinated); Tea; Tea (decaffeinated) |
| Less healthy foods | Fruit juices | Fruit juice |
|  | Refined grains | White bread; Biscuits; White pasta and rice; Oat cereal (sugar); Other cereal (sugar); Other bread |
|  | Potatoes | Mashed potatoes; Fried/roast potatoes; Potatoes and sweet potatoes (baked/boiled) |
|  | Sugar and non-sugar sweetened beverages | Sugar-sweetened beverages and other sugary drinks; Low/non-sugar sweetened beverages |
|  | Sweets and Desserts | Chocolate confectionery; Other sweets; Other desserts and cakes and pastries; Added sugars and preserves; Stewed fruit; Biscuit cereal; Soy desserts and yogurt |
| Animal food groups | Animal fat | Animal fat spread lower fat; Animal fat spread normal |
|  | Dairy | High fat cheese; Medium and low-fat cheese; Milk-based and powdered drinks; Full fat yogurt; Low fat yogurt; Whole milk; Semi skimmed milk; Skimmed milk and cholesterol-lowering milk; Milk-dairy desserts; Cream |
|  | Egg | Egg and egg dishes |
|  | Fish or Seafood | Oily fish; White fish and tinned tuna; Shellfish; Breaded/battered fish |
|  | Meat | Beef; Lamb; Other meat; Pork; Poultry; Processed meat; Breaded/battered chicken |
|  | Misc. animal-based foods | Pizza; Samosa; Pakora |
| Scoring method | The healthy Plant-Based Diet Index (hPDI) was calculated using repeated 24-hour dietary assessments from the UK Biobank. Participants with at least two valid dietary recalls were included, and the mean intake of each food item across available assessments was used to represent habitual diet. Implausible total energy intake was excluded using sex-specific cutoffs. Individual food items were then aggregated into 18 predefined food groups, including healthy plant foods, less healthy plant foods, and animal foods, based on the published UK Biobank food-group framework with minor adaptation to available variables. For most food groups, participants were ranked into sex-specific quintiles of intake. Healthy plant food groups were scored positively from 1 to 5, whereas less healthy plant food groups and animal food groups were reverse-scored from 5 to 1 for the hPDI. Miscellaneous animal foods were scored separately as 3, 2, or 1 points according to non-consumption, lower consumption, and higher consumption, respectively. The scores across all 18 food groups were summed to derive the final hPDI, with higher scores indicating greater adherence to a healthy plant-based dietary pattern. | |

^a^Food intakes were averaged across at least two valid Oxford WebQ assessments. Healthy plant foods were scored positively and less healthy plant foods and animal foods were reverse scored. The component scores were summed to derive the hPDI.

Supplementary Table S3. Calculation method for healthy plant-based diet-lifestyle score ^a^.

|  | Method of measurement | Quantification | |
| --- | --- | --- | --- |
| hPDI | Measurement: web-based 24-hour dietary recalls administered at least twice. | Quantiles of hPDI score | |
|  |  | Scoring:  Points  100  80  50  25  0 | Quantile  ≥ 95^th^ percentile (top/ideal diet)  75^th^-94^th^ percentile  50^th^-74^th^ percentile  25^th^-49^th^ percentile  1^st^-24^th^ percentile (bottom/ least ideal quartile) |
| Physical activity | Measurement: MET minutes per week for moderate and vigorous activity. | MET hours per week | |
|  |  | Scoring:  Points  100  90  80  60  40  20  0 | MET hours/week  ≥ 7·5  6-7·45  4·5-5·95  3-4·45  1·5-2·95  0·05-1·45  0 |
| Smoking status | Measurement: Self-reported use of cigarettes or inhaled  NDS  Tool for measurement: questionnaires during home interviews. Example questions: “Current cigarette smoker?”, “Past cigarette smoker?”, “How old were you when you quit smoking cigarettes?” | Scoring:  Points  100  [75  50  25]  50  0 | Status  Never smoker  Former smoker, quit ≥5 y  Former smoker, quit 1-<5 y  Former smoker, quit <1 y  Former smoker  Current smoker |
| Sleep health | Measurement: Self-reported average hours of sleep per night  Tool for measurement: questionnaires during home interviews. Example questions: “How long did you usually sleep per night (in hours)?” | Scoring:  Points  100  90  70  40  20  0 | Level  7-<9  9-<10  6-<7  5-<6 or ≥10  4-<5  <4 |

^a^Percentile categories apply only to conversion of the total hPDI into the 0-100 dietary component score. The overall hPDI-Lifestyle score was the arithmetic mean of the diet, physical activity, smoking, and sleep component scores. Abbreviations: hPDI, healthy plant-based diet index; MET, metabolic equivalent task.

Supplementary Figure S1. Directed acyclic graph illustrating the causal framework for covariate selection in the association between the hPDI-Lifestyle score and obesity-related cancer risk ^a^.

^a^Arrows represent hypothesized causal relationships. BMI and diabetes may act as confounders or metabolic intermediates, or may result from long-term lifestyle behaviours. They were included in the further-adjusted Model 3. Abbreviations: BMI, body mass index; hPDI-Lifestyle score, healthy plant-based diet index-lifestyle score.

Supplementary Table S4. Associations of hPDI-Lifestyle score with incidence, all-cause mortality, and cause-specific mortality of obesity-related cancers after excluding cases diagnosed within the first 2 years of follow-up.

| hPDI-Lifestyle score | Hazard ratio (95% CI) ^a^ | P value |
| --- | --- | --- |
|  | Incidence of obesity-related cancers |  |
| T1 | Reference | -- |
| T2 | 0.83 (0.75, 0.92) | <0.001 |
| T3 | 0.81 (0.73, 0.91) | <0.001 |
| Per SD increment in hPDI-Lifestyle score | 0.91 (0.87, 0.95) | <0.001 |
|  | Obesity-related cancer mortality |  |
| T1 | Reference | -- |
| T2 | 0.77 (0.68, 0.89) | <0.001 |
| T3 | 0.67 (0.57, 0.79) | <0.001 |
| Per SD increment in hPDI-Lifestyle score | 0.83 (0.78, 0.88) | <0.001 |
|  | All-cause mortality |  |
| T1 | Reference | -- |
| T2 | 0.75 (0.71, 0.80) | <0.001 |
| T3 | 0.64 (0.60, 0.69) | <0.001 |
| Per SD increment in hPDI-Lifestyle score | 0.79 (0.77, 0.81) | <0.001 |

^a^Model was adjusted for BMI category, diabetes, total energy intake, alcohol consumption status, sex, ethnicity, education level, family history of cancer, and average total household income.

Abbreviations: hPDI-Lifestyle score, healthy plant-based diet index-lifestyle score; CI, confidence interval.

Supplementary Table S5. Associations of the hPDI-Lifestyle score with obesity-related cancer incidence, cause-specific mortality, and all-cause mortality using an alternative categorical classification of the hPDI-Lifestyle score ^a^.

| hPDI-Lifestyle score | Hazard ratio (95% CI) ^b^ | P value |
| --- | --- | --- |
|  | Incidence of obesity-related cancers |  |
| Poor (0-49) | Reference | -- |
| Intermediate (50-79) | 0.97 (0.83, 1.14) | 0.748 |
| Ideal (80-100) | 0.84 (0.72, 0.99) | 0.040 |
|  | Obesity-related cancer mortality |  |
| Poor (0-49) | Reference | -- |
| Intermediate (50-79) | 0.73 (0.61, 0.89) | 0.002 |
| Ideal (80-100) | 0.56 (0.46, 0.69) | <0.001 |
|  | All-cause mortality |  |
| Poor (0-49) | Reference | -- |
| Intermediate (50-79) | 0.75 (0.70, 0.80) | <0.001 |
| Ideal (80-100) | 0.64 (0.59, 0.69) | <0.001 |

^a^ The overall hPDI-Lifestyle score was classified as poor (0-49), intermediate (50-79), or ideal (80-100).

^b^ Model was adjusted for BMI category, diabetes, total energy intake, alcohol consumption status, sex, ethnicity, education level, family history of cancer, and average total household income.

Abbreviations: hPDI-Lifestyle score, healthy plant-based diet index-lifestyle score; CI, confidence interval.

Supplementary Table S6. Competing-risk analyses for the associations of the hPDI-Lifestyle score with incidence and cause-specific mortality of obesity-related cancers using Fine-Gray subdistribution hazard models.

| hPDI-Lifestyle score | Subdistribution HR (95% CI)^a^ | P value |
| --- | --- | --- |
|  | Incidence of obesity-related cancers |  |
| T1 | Reference | -- |
| T2 | 0.85 (0.77, 0.94) | 0.002 |
| T3 | 0.81 (0.73, 0.90) | <0.001 |
| Per SD increment in hPDI-Lifestyle score | 0.94 (0.90, 0.98) | 0.003 |
|  | Obesity-related cancers mortality |  |
| T1 | Reference | -- |
| T2 | 0.83 (0.72, 0.94) | 0.005 |
| T3 | 0.73 (0.62, 0.85) | <0.001 |
| Per SD increment in hPDI-Lifestyle score | 0.88 (0.83, 0.92) | <0.001 |

^a^ Model was adjusted for BMI category, diabetes, total energy intake, alcohol consumption status, sex, ethnicity, education level, family history of cancer, and average total household income.

Abbreviations: hPDI-Lifestyle score, healthy plant-based diet index-lifestyle score; CI, confidence interval.

Supplementary Table S7. Associations of the hPDI-Lifestyle score with obesity-related cancer incidence, cause-specific mortality, and all-cause mortality stratified by sex using an alternative categorical classification of the hPDI-Lifestyle score.

|  | hPDI-Lifestyle score | Hazard ratio (95% CI)^a^ | P value |
| --- | --- | --- | --- |
|  |  | Incidence of obesity-related cancers |  |
| Female | Poor (0-49) | Reference | -- |
|  | Intermediate (50-79) | 0.99 (0.78, 1.25) | 0.928 |
|  | Ideal (80-100) | 0.97 (0.76, 1.24) | 0.813 |
|  | Per SD increment in hPDI-Lifestyle score | 0.97 (0.92, 1.03) | 0.375 |
| Male | Poor (0-49) | Reference | -- |
|  | Intermediate (50-79) | 0.97 (0.78, 1.19) | 0.750 |
|  | Ideal (80-100) | 0.72 (0.58, 0.91) | 0.005 |
|  | Per SD increment in hPDI-Lifestyle score | 0.87 (0.82, 0.92) | <0.001 |
|  | P for interaction |  | 0.020 |
|  |  | Obesity-related cancer mortality |  |
| Female | Poor (0-49) | Reference | -- |
|  | Intermediate (50-79) | 0.73 (0.53, 0.99) | 0.046 |
|  | Ideal (80-100) | 0.55 (0.40, 0.76) | <0.001 |
|  | Per SD increment in hPDI-Lifestyle score | 0.86 (0.79, 0.94) | <0.001 |
| Male | Poor (0-49) | Reference | -- |
|  | Intermediate (50-79) | 0.69 (0.53, 0.89) | 0.005 |
|  | Ideal (80-100) | 0.53 (0.40, 0.70) | <0.001 |
|  | Per SD increment in hPDI-Lifestyle score | 0.81 (0.75, 0.87) | <0.001 |
|  | P for interaction |  | 0.862 |
|  |  | All-cause mortality |  |
| Female | Poor (0-49) | Reference | -- |
|  | Intermediate (50-79) | 0.55 (0.48, 0.62) | <0.001 |
|  | Ideal (80-100) | 0.42 (0.37, 0.49) | <0.001 |
|  | Per SD increment in hPDI-Lifestyle score | 0.76 (0.72, 0.79) | <0.001 |
| Male | Poor (0-49) | Reference | -- |
|  | Intermediate (50-79) | 0.64 (0.58, 0.71) | <0.001 |
|  | Ideal (80-100) | 0.50 (0.45, 0.56) | <0.001 |
|  | Per SD increment in hPDI-Lifestyle score | 0.80 (0.77, 0.82) | <0.001 |
|  | P for interaction |  | 0.168 |

^a^ Models were adjusted for BMI category, diabetes, total energy intake, alcohol consumption status, ethnicity, education level, family history of cancer, and average total household income.

Abbreviations: hPDI-Lifestyle score, healthy plant-based diet index-lifestyle score; CI, confidence interval.

Supplementary Table S8. Sex-stratified associations with obesity-related cancer incidence, cancer-specific mortality, and all-cause mortality after excluding events occurring within the first two years.

|  | hPDI-Lifestyle score | Hazard ratio (95% CI)^a^ | P value |
| --- | --- | --- | --- |
|  |  | Incidence of obesity-related cancers |  |
| Female | T1 | Reference | -- |
|  | T2 | 0.90 (0.78, 1.04) | 0.137 |
|  | T3 | 0.93 (0.79, 1.08) | 0.328 |
|  | Per SD increment | 0.96 (0.90, 1.02) | 0.173 |
| Male | T1 | Reference | -- |
|  | T2 | 0.76 (0.66, 0.88) | <0.001 |
|  | T3 | 0.72 (0.61, 0.85) | <0.001 |
|  | Per SD increment | 0.87 (0.82, 0.93) | <0.001 |
|  | P for interaction |  | 0.106 |
|  |  | Cause-specific mortality of obesity-related cancers |  |
| Female | T1 | Reference | -- |
|  | T2 | 0.86 (0.70, 1.05) | 0.144 |
|  | T3 | 0.76 (0.60, 0.95) | 0.017 |
|  | Per SD increment | 0.84 (0.77, 0.92) | <0.001 |
| Male | T1 | Reference | -- |
|  | T2 | 0.70 (0.58, 0.85) | <0.001 |
|  | T3 | 0.63 (0.50, 0.79) | <0.001 |
|  | Per SD increment | 0.81 (0.75, 0.88) | <0.001 |
|  | P for interaction |  | 0.163 |
|  |  | All-cause mortality |  |
| Female | T1 | Reference | -- |
|  | T2 | 0.70 (0.63, 0.78) | <0.001 |
|  | T3 | 0.62 (0.56, 0.70) | <0.001 |
|  | Per SD increment | 0.76 (0.73, 0.79) | <0.001 |
| Male | T1 | Reference | -- |
|  | T2 | 0.77 (0.71, 0.84) | <0.001 |
|  | T3 | 0.65 (0.59, 0.71) | <0.001 |
|  | Per SD increment | 0.80 (0.77, 0.83) | <0.001 |
|  | P for interaction |  | 0.459 |

^a^ Models were adjusted for BMI category, diabetes, total energy intake, alcohol consumption status, ethnicity, education level, family history of cancer, and average total household income.

Abbreviations: hPDI-Lifestyle score, healthy plant-based diet index-lifestyle score; CI, confidence interval.

Supplementary Table S9. Associations of the hPDI-Lifestyle score with obesity-related cancer incidence, obesity-related cancer-specific mortality, and all-cause mortality after multiple imputation.

| hPDI-Lifestyle score | Events/participants | Hazard ratio (95% CI) ^a^ | P value |
| --- | --- | --- | --- |
|  | 2537/104713 | Incidence of obesity-related cancers |  |
| T1 |  | Reference |  |
| T2 |  | 0.87 (0.79, 0.95) | 0.002 |
| T3 |  | 0.81 (0.73, 0.90) | <0.001 |
| Per SD increment |  | 0.91 (0.88, 0.95) | <0.001 |
|  | 1180/104713 | Obesity-related cancers mortality |  |
| T1 |  | Reference |  |
| T2 |  | 0.74 (0.65, 0.84) | <0.001 |
| T3 |  | 0.64 (0.55, 0.75) | <0.001 |
| Per SD increment |  | 0.81 (0.76, 0.85) | <0.001 |
|  | 7081/104713 | All-cause mortality |  |
| T1 |  | Reference |  |
| T2 |  | 0.73 (0.69, 0.77) | <0.001 |
| T3 |  | 0.64 (0.60, 0.68) | <0.001 |
| Per SD increment |  | 0.78 (0.76, 0.80) | <0.001 |

^a^Model was adjusted for BMI category, diabetes, total energy intake, alcohol consumption status, sex, ethnicity, education level, family history of cancer, and average total household income.

Abbreviations: hPDI-Lifestyle score, healthy plant-based diet index-lifestyle score; CI, confidence interval.

Multiple imputation was performed for missing covariates using 10 imputed datasets; exposure, outcome, follow-up time, age, sex, and total energy intake were not imputed. Cox estimates were pooled using Rubin’s rules.

Supplementary Table S10. Numbers of participants, events, person-years, and event rates per 1,000 person-years across hPDI-Lifestyle score tertiles for obesity-related cancer incidence, obesity-related cancer mortality, and all-cause mortality ^a^.

| Outcome | hPDI-Lifestyle score tertile | Events /participants | Person-years | Rate per 1,000  person-years |
| --- | --- | --- | --- | --- |
| Obesity-related cancer incidence | T1 | 913/33220 | 524093.7 | 1.74 |
|  | T2 | 785/34394 | 547467.1 | 1.43 |
|  | T3 | 560/26149 | 418043.4 | 1.34 |
| Obesity-related cancer mortality | T1 | 474/33220 | 490658.8 | 0.97 |
|  | T2 | 373/34394 | 511653.6 | 0.73 |
|  | T3 | 248/26149 | 390542.1 | 0.64 |
| All-cause mortality | T1 | 2717/33220 | 490658.8 | 5.54 |
|  | T2 | 2115/34394 | 511653.6 | 4.13 |
|  | T3 | 1383/26149 | 390542.1 | 3.54 |

Event rates were calculated as the number of events divided by person-years and multiplied by 1,000. Participant numbers and follow-up time are outcome specific.

Abbreviations: hPDI-Lifestyle score, healthy plant-based diet index-lifestyle score.

Supplementary Table S11. Comparison of baseline characteristics between participants included in and excluded from the eligible cohort ^a^.

| Characteristic | Level | Excluded  N=397,243 (%) | Included N=104,713(%) | P value |
| --- | --- | --- | --- | --- |
| Sex | Women | 216,748 (54.6) | 56,300 (53.8) | <0.001 |
|  | Men | 180,495 (45.4) | 48,413 (46.2) |  |
|  | Missing | 0 (0.0) | 0 (0.0) |  |
| Age, years | Mean (SD) | 56.7 (8.1) | 55.9 (7.9) | <0.001 |
|  | Missing | 0 (0.0) | 0 (0.0) |  |
| Education | College/university degree | 109,536 (28.3) | 51,401 (49.2) | <0.001 |
|  | Other qualifications | 198,318 (51.2) | 47,360 (45.3) |  |
|  | None | 79,473 (20.5) | 5,749 (5.5) |  |
|  | Missing | 9,916 (2.5) | 203 (0.2) |  |
| Ethnicity | White | 370,994 (94.0) | 101,199 (96.9) | <0.001 |
|  | Mixed | 2,382 (0.6) | 567 (0.5) |  |
|  | Asian | 10,170 (2.6) | 1,270 (1.2) |  |
|  | Black | 7,237 (1.8) | 811 (0.8) |  |
|  | Other | 3,960 (1.0) | 592 (0.6) |  |
|  | Missing | 2,500 (0.6) | 274 (0.3) |  |
| Household income | <£18,000 | 84,618 (25.8) | 12,510 (12.9) | <0.001 |
|  | £18,000–30,999 | 85,750 (26.1) | 22,322 (23.0) |  |
|  | £31,000–51,999 | 82,503 (25.2) | 28,150 (29.0) |  |
|  | £52,000–100,000 | 60,439 (18.4) | 25,716 (26.5) |  |
|  | >£100,000 | 14,695 (4.5) | 8,209 (8.5) |  |
|  | Missing | 69,238 (17.4) | 7,806 (7.5) |  |
| Alcohol consumption status | Never | 19,554 (4.9) | 2,800 (2.7) | <0.001 |
|  | Previous | 15,141 (3.8) | 2,939 (2.8) |  |
|  | Current | 360,928 (91.2) | 98,942 (94.5) |  |
|  | Missing | 1,620 (0.4) | 32 (0.0) |  |
| Smoking status | Never | 213,358 (54.1) | 59,876 (57.2) | <0.001 |
|  | Previous | 135,276 (34.3) | 37,567 (35.9) |  |
|  | Current | 45,661 (11.6) | 7,270 (6.9) |  |
|  | Missing | 2,948 (0.7) | 0 (0.0) |  |
| Sleep duration, hours/day | Median (IQR) | 7.0 (6.0–8.0) | 7.0 (7.0–8.0) | <0.001 |
|  | Missing | 4,210 (1.1) | 0 (0.0) |  |
| Vigorous activity, MET-min/week | Median (IQR) | 240.0 (0.0–960.0) | 240.0 (0.0–960.0) | <0.001 |
|  | Missing | 117,103 (29.5) | 0 (0.0) |  |
| BMI category | Underweight | 2,024 (0.5) | 600 (0.6) | <0.001 |
|  | Normal | 120,906 (30.7) | 41,286 (39.5) |  |
|  | Overweight | 168,947 (42.8) | 42,950 (41.1) |  |
|  | Obesity | 102,484 (26.0) | 19,656 (18.8) |  |
|  | Missing | 2,882 (0.7) | 221 (0.2) |  |
| Central obesity | No | 266,517 (67.4) | 78,477 (75.0) | <0.001 |
|  | Yes | 128,692 (32.6) | 26,111 (25.0) |  |
|  | Missing | 2,034 (0.5) | 125 (0.1) |  |
| Diabetes | No | 375,648 (94.7) | 101,041 (96.5) | <0.001 |
|  | Yes | 20,940 (5.3) | 3,637 (3.5) |  |
|  | Missing | 655 (0.2) | 35 (0.0) |  |
| Family history of cancer | No | 292,939 (77.8) | 77,826 (76.5) | <0.001 |
|  | Yes | 83,578 (22.2) | 23,959 (23.5) |  |
|  | Missing | 20,726 (5.2) | 2,928 (2.8) |  |

^a^ Values are presented as n (%) for categorical variables, mean (SD) for age, and median (IQR) for sleep duration and vigorous physical activity. Percentages for categorical variables were calculated among participants with non-missing data, with missing values reported separately. Differences represent the included group minus the excluded group and are expressed as percentage points (pp) for categorical variables. P values were calculated using Pearson’s chi-squared test for categorical variables, Welch’s t-test for age, and the Wilcoxon rank-sum test for sleep duration and vigorous physical activity.

Supplementary Table S12. Distribution of the four component scores of the hPDI-Lifestyle score ^a^.

| Component | Mean (SD) | Median (IQR) | Score ≥80, n (%) | Score =100, n (%) |
| --- | --- | --- | --- | --- |
| Diet | 42.9 (31.4) | 50 (25–80) | 29,643 (28.3) | 7,054 (6.7) |
| Physical activity | 79.8 (35.7) | 100 (80–100) | 78,903 (75.4) | 72,988 (69.7) |
| Smoking | 80.4 (28.6) | 100 (75–100) | 59,876 (57.2) | 59,876 (57.2) |
| Sleep | 91.3 (16.5) | 100 (90–100) | 81,323 (77.7) | 76,011 (72.6) |

^a^ Values were calculated among 104,713 participants with complete data for all four component scores. SD, standard deviation; IQR, interquartile range.

**Supplementary Table S13. Correlations among the four component scores and the overall hPDI-Lifestyle score ^a^.**

| Component | Diet | Physical activity | Smoking | Sleep |
| --- | --- | --- | --- | --- |
| Diet | 1.000 | 0.079*** | −0.007* | 0.010** |
| Physical activity | 0.079*** | 1.000 | 0.017*** | 0.047*** |
| Smoking | −0.007* | 0.017*** | 1.000 | 0.031*** |
| Sleep | 0.010** | 0.047*** | 0.031*** | 1.000 |
| hPDI-lifestyle score | 0.599*** | 0.587*** | 0.425*** | 0.308*** |

^a^ Values are Spearman correlation coefficients calculated among 104,713 participants.

* P<0.05; ** P<0.01; *** P<0.001.

Supplementary Table S14. Association between the number of ideal health behaviours and obesity-related cancer incidence.

| Variable | Hazard ratio (95% CI) ^a^ | P value |
| --- | --- | --- |
| 1 vs 0 healthy behaviours | 1.07 (0.88, 1.31) | 0.504 |
| 2 vs 0 healthy behaviours | 0.89 (0.73, 1.08) | 0.249 |
| 3 vs 0 healthy behaviours | 0.80 (0.65, 0.98) | 0.035 |
| 4 vs 0 healthy behaviours | 0.67 (0.45, 0.99) | 0.042 |
| Per additional healthy behaviour | 0.89 (0.85, 0.93) | <0.001 |

^a^ Models were adjusted for BMI category, diabetes, total energy intake, alcohol consumption status, sex, ethnicity, education, family history of cancer, and household income.

Each behaviour was considered ideal only when its component score was 100. The ≥80 threshold was not used because its resulting reference group was small and had few events, producing unstable risk estimates.

Supplementary Table S15. Component-specific, mutually adjusted, and smoking-excluded associations of the hPDI-Lifestyle score components with obesity-related cancer incidence and mortality outcomes.

| Outcome | Model | Component score | HR per SD increment (95% CI) ^a^ | P value |
| --- | --- | --- | --- | --- |
| Obesity-related cancer incidence | Component-specific | hPDI | 0.99 (0.95, 1.03) | 0.609 |
|  |  | Physical activity | 0.93 (0.89, 0.96) | <0.001 |
|  |  | Smoking | 0.94 (0.90, 0.98) | 0.003 |
|  |  | Sleep | 1.00 (0.96, 1.04) | 0.961 |
|  | Mutually adjusted | hPDI | 1.00 (0.95, 1.04) | 0.823 |
|  |  | Physical activity | 0.93 (0.89, 0.97) | <0.001 |
|  |  | Smoking | 0.94 (0.90, 0.98) | 0.004 |
|  |  | Sleep | 1.00 (0.96, 1.05) | 0.859 |
|  | Excluding smoking | hPDI, physical activity, and sleep | 0.94 (0.90, 0.98) | 0.005 |
| Obesity-related cancer-specific mortality | Component-specific | hPDI | 0.93 (0.87, 0.99) | 0.018 |
|  |  | Physical activity | 0.90 (0.85, 0.95) | <0.001 |
|  |  | Smoking | 0.83 (0.78, 0.88) | <0.001 |
|  |  | Sleep | 0.96 (0.91, 1.02) | 0.160 |
|  | Mutually adjusted | hPDI | 0.94 (0.88, 1.00) | 0.043 |
|  |  | Physical activity | 0.91 (0.86, 0.96) | 0.001 |
|  |  | Smoking | 0.83 (0.79, 0.88) | <0.001 |
|  |  | Sleep | 0.97 (0.91, 1.02) | 0.247 |
|  | Excluding smoking | hPDI, physical activity, and sleep | 0.88 (0.83, 0.94) | <0.001 |
| All-cause mortality | Component-specific | hPDI | 0.93 (0.90, 0.95) | <0.001 |
|  |  | Physical activity | 0.88 (0.86, 0.90) | <0.001 |
|  |  | Smoking | 0.81 (0.79, 0.83) | <0.001 |
|  |  | Sleep | 0.92 (0.90, 0.95) | <0.001 |
|  | Mutually adjusted | hPDI | 0.94 (0.91, 0.96) | <0.001 |
|  |  | Physical activity | 0.89 (0.87, 0.91) | <0.001 |
|  |  | Smoking | 0.81 (0.80, 0.83) | <0.001 |
|  |  | Sleep | 0.93 (0.91, 0.95) | <0.001 |
|  | Excluding smoking | hPDI, physical activity, and sleep | 0.86 (0.84, 0.88) | <0.001 |

^a^All models were additionally adjusted for BMI category, diabetes, total energy intake, alcohol consumption status, sex, ethnicity, education, family history of cancer, and household income. Hazard ratios are expressed per SD increment. Component-specific models included one component at a time; mutually adjusted models included all four components simultaneously. The smoking-excluded score comprised hPDI, physical activity, and sleep, with smoking score included as a covariate.

Abbreviations: CI, confidence interval; hPDI, healthy plant-based diet index; HR, hazard ratio; SD, standard deviation.

Supplementary Table S16. Site-specific associations of the hPDI-Lifestyle score with obesity-related cancer incidence.

| Cancer site | Events/participants | Variable | Hazard ratio (95% CI) ^a^ | P value |
| --- | --- | --- | --- | --- |
| Oesophagus (C15) | 163/93,763 | T1 | Reference | — |
|  |  | T2 | 0.50 (0.35, 0.73) | <0.001 |
|  |  | T3 | 0.55 (0.37, 0.83) | 0.004 |
|  |  | Per SD increment | 0.78 (0.67, 0.90) | <0.001 |
| Gastric cardia (C16.0) | 48/93,763 | Not estimated | — | — |
| Colorectum (C18, C19.9, C20.9) | 767/93,763 | T1 | Reference | — |
|  |  | T2 | 0.88 (0.74, 1.03) | 0.120 |
|  |  | T3 | 0.80 (0.67, 0.97) | 0.021 |
|  |  | Per SD increment | 0.92 (0.85, 0.98) | 0.016 |
| Liver (C22) | 89/93,763 | T1 | Reference | — |
|  |  | T2 | 0.98 (0.61, 1.56) | 0.929 |
|  |  | T3 | 0.71 (0.39, 1.28) | 0.253 |
|  |  | Per SD increment | 0.79 (0.65, 0.96) | 0.020 |
| Gallbladder (C23) | 11/93,763 | Not estimated | — | — |
| Pancreas (C25) | 235/93,763 | T1 | Reference | — |
|  |  | T2 | 0.66 (0.48, 0.89) | 0.006 |
|  |  | T3 | 0.69 (0.50, 0.97) | 0.030 |
|  |  | Per SD increment | 0.84 (0.74, 0.95) | 0.007 |
| Kidney (C64) | 248/93,763 | T1 | Reference | — |
|  |  | T2 | 0.92 (0.69, 1.23) | 0.568 |
|  |  | T3 | 0.83 (0.60, 1.15) | 0.260 |
|  |  | Per SD increment | 0.93 (0.82, 1.05) | 0.254 |
| Corpus uteri (C54–C55) | 286/49,593 | T1 | Reference | — |
|  |  | T2 | 1.22 (0.92, 1.61) | 0.163 |
|  |  | T3 | 1.25 (0.92, 1.70) | 0.154 |
|  |  | Per SD increment | 1.13 (1.00, 1.28) | 0.049 |
| Ovary (C56) | 160/49,593 | T1 | Reference | — |
|  |  | T2 | 0.80 (0.56, 1.15) | 0.225 |
|  |  | T3 | 0.77 (0.51, 1.15) | 0.207 |
|  |  | Per SD increment | 0.89 (0.76, 1.04) | 0.148 |
| Thyroid (C73) | 68/93,763 | T1 | Reference | — |
|  |  | T2 | 1.21 (0.69, 2.12) | 0.508 |
|  |  | T3 | 1.15 (0.61, 2.17) | 0.662 |
|  |  | Per SD increment | 1.02 (0.80, 1.31) | 0.860 |
| Malignant neoplasm of the meninges (C70) | 1/93,763 | Not estimated | — | — |
| Multiple myeloma (C90.0) | 180/93,763 | T1 | Reference | — |
|  |  | T2 | 0.85 (0.60, 1.20) | 0.346 |
|  |  | T3 | 0.84 (0.58, 1.23) | 0.370 |
|  |  | Per SD increment | 0.96 (0.83, 1.11) | 0.583 |

^a^Models were adjusted for BMI category, diabetes, total energy intake, alcohol consumption status, sex (except female-specific analyses), ethnicity, education, family history of cancer, and household income. Association estimates were calculated only for cancer sites with at least 50 events; sites with fewer than 50 events are shown as not estimated. Corpus uteri and ovarian cancers were analysed among women only.

Abbreviations: CI, confidence interval; HR, hazard ratio; SD, standard deviation.

Supplementary Table S17. Leave-one-cancer-site-out associations of the hPDI-Lifestyle score with obesity-related cancer incidence.

| Excluded cancer site | Events/participants | Variable | Hazard ratio (95% CI) ^a^ | P value |
| --- | --- | --- | --- | --- |
| Oesophagus (C15) | 2,097/93,763 | T1 | Reference | — |
|  |  | T2 | 0.89 (0.81, 0.99) | 0.027 |
|  |  | T3 | 0.85 (0.76, 0.95) | 0.004 |
|  |  | Per SD increment | 0.93 (0.89, 0.97) | 0.001 |
| Gastric cardia (C16.0) | 2,210/93,763 | T1 | Reference | — |
|  |  | T2 | 0.86 (0.78, 0.94) | 0.002 |
|  |  | T3 | 0.82 (0.73, 0.91) | <0.001 |
|  |  | Per SD increment | 0.92 (0.88, 0.96) | <0.001 |
| Colorectum (C18, C19.9, C20.9) | 1,492/93,763 | T1 | Reference | — |
|  |  | T2 | 0.85 (0.75, 0.96) | 0.007 |
|  |  | T3 | 0.83 (0.73, 0.95) | 0.007 |
|  |  | Per SD increment | 0.92 (0.88, 0.97) | 0.002 |
| Liver (C22) | 2,170/93,763 | T1 | Reference | — |
|  |  | T2 | 0.85 (0.77, 0.94) | 0.002 |
|  |  | T3 | 0.83 (0.74, 0.92) | <0.001 |
|  |  | Per SD increment | 0.93 (0.89, 0.97) | <0.001 |
| Gallbladder (C23) | 2,247/93,763 | T1 | Reference | — |
|  |  | T2 | 0.86 (0.78, 0.94) | 0.002 |
|  |  | T3 | 0.82 (0.74, 0.91) | <0.001 |
|  |  | Per SD increment | 0.92 (0.88, 0.96) | <0.001 |
| Pancreas (C25) | 2,023/93,763 | T1 | Reference | — |
|  |  | T2 | 0.89 (0.80, 0.98) | 0.020 |
|  |  | T3 | 0.84 (0.75, 0.94) | 0.003 |
|  |  | Per SD increment | 0.93 (0.89, 0.97) | 0.001 |
| Kidney (C64) | 2,010/93,763 | T1 | Reference | — |
|  |  | T2 | 0.85 (0.77, 0.94) | 0.002 |
|  |  | T3 | 0.82 (0.73, 0.92) | <0.001 |
|  |  | Per SD increment | 0.92 (0.88, 0.96) | <0.001 |
| Corpus uteri (C54–C55) | 1,973/93,763 | T1 | Reference | — |
|  |  | T2 | 0.82 (0.74, 0.91) | <0.001 |
|  |  | T3 | 0.77 (0.69, 0.87) | <0.001 |
|  |  | Per SD increment | 0.89 (0.86, 0.93) | <0.001 |
| Ovary (C56) | 2,098/93,763 | T1 | Reference | — |
|  |  | T2 | 0.86 (0.78, 0.95) | 0.004 |
|  |  | T3 | 0.83 (0.74, 0.92) | <0.001 |
|  |  | Per SD increment | 0.92 (0.88, 0.96) | <0.001 |
| Thyroid (C73) | 2,190/93,763 | T1 | Reference | — |
|  |  | T2 | 0.85 (0.77, 0.94) | 0.001 |
|  |  | T3 | 0.81 (0.73, 0.91) | <0.001 |
|  |  | Per SD increment | 0.92 (0.88, 0.96) | <0.001 |
| Malignant neoplasm of the meninges (C70) | 2,257/93,763 | T1 | Reference | — |
|  |  | T2 | 0.86 (0.78, 0.95) | 0.002 |
|  |  | T3 | 0.82 (0.74, 0.92) | <0.001 |
|  |  | Per SD increment | 0.92 (0.88, 0.96) | <0.001 |
| Multiple myeloma (C90.0) | 2,078/93,763 | T1 | Reference | — |
|  |  | T2 | 0.86 (0.78, 0.95) | 0.003 |
|  |  | T3 | 0.82 (0.73, 0.92) | <0.001 |
|  |  | Per SD increment | 0.92 (0.88, 0.96) | <0.001 |

^a^Models were adjusted for BMI category, diabetes, total energy intake, alcohol consumption status, sex, ethnicity, education, family history of cancer, and household income. Each model excluded events from the cancer site indicated while retaining all participants at risk. Events denote the remaining composite-outcome events after that exclusion.

Abbreviations: CI, confidence interval; HR, hazard ratio; SD, standard deviation.

Supplementary Table S18. Sex-stratified associations of the hPDI-Lifestyle score with incidence and cancer-specific mortality of obesity-related cancers occurring in both sexes.

| Sex | hPDI-Lifestyle score | Events/participants | Hazard ratio (95% CI)ᵃ | P value |
| --- | --- | --- | --- | --- |
|  |  | 726/49,593 | Incidence of obesity-related cancers |  |
| Female | T1 |  | Reference | -- |
|  | T2 |  | 0.90 (0.76, 1.07) | 0.216 |
|  | T3 |  | 0.90 (0.74, 1.08) | 0.254 |
|  | Per SD increment |  | 0.94 (0.87, 1.01) | 0.105 |
|  |  | 1,084/44,170 |  |  |
| Male | T1 |  | Reference | -- |
|  | T2 |  | 0.78 (0.68, 0.89) | <0.001 |
|  | T3 |  | 0.71 (0.60, 0.83) | <0.001 |
|  | Per SD increment |  | 0.87 (0.82, 0.92) | <0.001 |
|  | P for interaction |  |  | 0.127 |
|  |  | 360/49,593 | Obesity-related cancer-specific mortality |  |
| Female | T1 |  | Reference | -- |
|  | T2 |  | 0.80 (0.63, 1.02) | 0.075 |
|  | T3 |  | 0.77 (0.59, 1.00) | 0.054 |
|  | Per SD increment |  | 0.83 (0.74, 0.92) | <0.001 |
|  |  | 583/44,170 |  |  |
| Male | T1 |  | Reference | -- |
|  | T2 |  | 0.68 (0.56, 0.82) | <0.001 |
|  | T3 |  | 0.59 (0.47, 0.73) | <0.001 |
|  | Per SD increment |  | 0.79 (0.74, 0.86) | <0.001 |
|  | P for interaction |  |  | 0.128 |

^a^Sex-stratified models were adjusted for BMI category, diabetes, total energy intake, alcohol consumption status, ethnicity, education, family history of cancer, and household income. The composite outcome was restricted to cancer sites occurring in both sexes by excluding corpus uteri and ovarian cancers.

Abbreviations: CI, confidence interval; hPDI-Lifestyle score, healthy plant-based diet index-lifestyle score; HR, hazard ratio; SD, standard deviation.
